# (Bio)Degradable Polymeric Materials for Sustainable Future—Part 3: Degradation Studies of the PHA/Wood Flour-Based Composites and Preliminary Tests of Antimicrobial Activity

**DOI:** 10.3390/ma13092200

**Published:** 2020-05-11

**Authors:** Marta Musioł, Sebastian Jurczyk, Michał Sobota, Magdalena Klim, Wanda Sikorska, Magdalena Zięba, Henryk Janeczek, Joanna Rydz, Piotr Kurcok, Brian Johnston, Izabela Radecka

**Affiliations:** 1 Centre of Polymer and Carbon Materials, Polish Academy of Sciences, 34. M. Curie-Sklodowska St., 41-819 Zabrze, Poland; m.sobota@cmpw-pan.edu.pl (M.S.); klim.magdalena@gmail.com (M.K.); wsikorska@cmpw-pan.edu.pl (W.S.); mzieba@cmpw-pan.edu.pl (M.Z.); hjaneczek@cmpw-pan.edu.pl (H.J.); jrydz@cmpw-pan.edu.pl (J.R.); p.kurcok@cmpw-pan.edu.pl (P.K.); 2 Łukasieiwcz Research Network – Institute for Engineering of Polymer Materials and Dyes, 55, M. Sklodowska-Curie St., 87-100 Toruń, Poland; s.jurczyk@impib.pl; 3 Department of Microbiology and Virology, School of Pharmacy with the Division of Laboratory Medicine, Medical University of Silesia, 4 Jagiellońska St., 41-200 Sosnowiec, Poland; 4 Wolverhampton School of Sciences, Faculty of Science and Engineering, University of Wolverhampton, Wulfruna Street, Wolverhampton WV1 1LY, UK; B.Johnston@wlv.ac.uk (B.J.); I.Radecka@wlv.ac.uk (I.R.)

**Keywords:** biodegradable composites, PHA, wood flour, biodegradation, organic recycling

## Abstract

The need for a cost reduction of the materials derived from (bio)degradable polymers forces research development into the formation of biocomposites with cheaper fillers. As additives can be made using the post-consumer wood, generated during wood products processing, re-use of recycled waste materials in the production of biocomposites can be an environmentally friendly way to minimalize and/or utilize the amount of the solid waste. Also, bioactive materials, which possess small amounts of antimicrobial additives belong to a very attractive packaging industry solution. This paper presents a study into the biodegradation, under laboratory composting conditions, of the composites that consist of poly[(*R*)-3-hydroxybutyrate-*co*-4-hydroxybutyrate)] and wood flour as a polymer matrix and natural filler, respectively. Thermogravimetric analysis, differential scanning calorimetry and scanning electron microscopy were used to evaluate the degradation progress of the obtained composites with different amounts of wood flour. The degradation products were characterized by multistage electrospray ionization mass spectrometry. Also, preliminary tests of the antimicrobial activity of selected materials with the addition of nisin were performed. The obtained results suggest that the different amount of filler has a significant influence on the degradation profile.

## 1. Introduction

The waste of products from conventional plastics is one of the most troublesome categories of refuse, which is a serious threat to the environment. An interesting alternative for conventional plastics may be bioplastics, including (bio)degradable polyesters such as polyhydroxyalkanoates (PHA)s or polylactide (PLA) [1,2]. The new polymeric materials used, e.g., in the packaging industries, maintain the functional properties of traditional plastics, but are also susceptible to biodegradation. To reduce the high price of (bio)degradable polymers and to improve their physical properties, biocomposites with natural, eco-friendly additives were prepared [3,4,5]. Wood flour is a material with low density, high stiffness, biodegradability and it is the most widespread natural, renewable and easily available material at low cost. Wood flour, a waste from woodworking and furniture companies, contains organic compounds such as cellulose, hemicellulose, and lignin. In addition, it also contains simple carbohydrates, proteins, starch, tannins, essential oils, natural rubber and mineral salts. The chemical composition of wood flour depends on the type of tree, climate and soil. So, composting should be the most appropriate method to dispose of this type of material [6]. The increase of customer awareness in the field of environmental responsibility concerning the recycling systems and the reuse techniques of solid waste materials, still need to achieve some developmental goals. All the possibilities of using new products, including composites with the addition of recycled wood, help to reduce the environmental impact and the consumption of the conventional polymers [7].

The European Commission (in 2019) presented a directive on the reduction of the impact of conventional plastic products on the environment, whose primary task is to achieve the environmental objectives set out in the Plastics Strategy in Europe. The basic intention underlined in both documents is a significant limitation, and for some products, even the elimination from the market of disposable items made of plastics, such as plastic cutlery, straws, plates, or ear sticks, from 2021. The Commission justifies this with the need to protect primarily the seas and oceans against the increasing pollution of plastics and the many consequences that result from it. The conventional plastics are a very useful material, but its use has become too common and irresponsible. Disposable products, due to their very short life cycle, should be replaced by more balanced counterparts, e.g., reusable or made from biodegradable materials [8].

The scientific interests in biocomposites containing wood flour have been observed for a few years. The Chiellini group investigated composites of plasticized poly(3-hydroxybutyrate) with beech wood flour, especially its thermal stability depending on the presence of plasticizer [9]. The environmentally friendly composites were obtained from pineapple fibers and poly[(*R*)-3-hydroxybutyrate-*co*-(*R*)-3-hydroxyvalerate] (PHBV), and the influence of the arrangement and orientation of the fibers in the polymer matrix on the composite properties were studied [10]. In the work on PLA-based biocomposites containing maple wood flour, authors concluded that the best mechanical properties were obtained at 40 wt. % of the additive. Moreover, authors suggested that, for improving of thermal and mechanical properties as well better fiber-matrix binding was responsible the polar structure of PLA [11]. Recently the biocomposites with improving mechanical strength contained wood flour (cellulose-based waste as filler) was investigated [12]. The nanocomposites containing the modified wood cellulose fibers and/or additives with antimicrobial activity were also applied in the production of environmentally friendly food packaging [13,14,15]. Generally, the authors highlighted the need to replace the non-degradable matrix in traditional wood-plastic composites with (bio)degradable polymers like PHAs or PLA [16]. The waste of such biocomposites, after their final use, can be disposed in the composting process [17]. The composting under industrial conditions (organic recycling) is the best way of biodegradable material utilization. The biodegradation study conducted under these conditions was a terrestrial mesocosms experiment, because it reflected the real conditions prevailing during this process. Biodegradation carried out under laboratory composting conditions, using a Micro-Oxymax respirometer, was a microcosm experiment and it was carried out as a reference test [18,19,20,21,22].

In packaging applications, identification of the interactions between the biocomposite components and package content helps to prevent of damage before, during and after the use of the final product. In addition, the wider applications of biocomposites require specific characterization of the properties of the (bio)degradable polymers, as well as optimization of the production of final goods, their processing and the recycling of post-consumer waste. Connecting these different elements together under the common thread of Forensic Engineering Studies of Advanced Polymeric Materials constitutes the novelty of this approach and provides a central driving force for the otherwise disconnected works. Such a methodology should help to design novel (bio)degradable, polymeric materials and it would avoid some of the failures of the commercial products manufactured from them [23,24,25,26,27,28]. The trends concerning the idea of smart packages are in line with the idea of a sustainable future [29]. Biodegradable bioactive composites, which possess some amount of antimicrobial additives, belong to very attractive eco-friendly materials. The antimicrobial properties of the biocomposites can be achieved by the incorporation of nisin. This bacteriocin shows a broad-spectrum of activity against Gram-positive bacterial strains and it is non-toxic and heat stable [30]. Shiroodi et al. investigated the PLA films with nisin as an additive. The obtained materials were considered an appropriate (bio)degradable film for food products that inherently extended shelf life [31].

This work deals with the problem of organic recycling of the materials containing the wood flour. The utilization of waste from the wood industry for the production of polyester composites was proposed. The characterization and biodegradation under composting conditions of composites made of poly[(*R*)-3-hydroxybutyrate-*co*-(*R*)-4-hydroxybutyrate] (P(3HB-*co*-4HB)) containing 10, 20, and 30 wt. % of wood flour were presented. To evaluate the degradation progress of the obtained composites with different amounts of wood flour the thermogravimetric analysis, differential scanning calorimetry and scanning electron microscope were used. Additionally, abiotic degradation tests in distilled water and buffer were carried out under laboratory conditions to verify the influence on the degradation rate of composting conditions. The characterization of degradation products with the aid of multistage electrospray ionization mass spectrometry (ESI–MS^n^) analysis was also performed. Moreover, the antimicrobial properties of P(3HB-*co*-4HB) with 5 wt. % additive of nisin have also been determined.

## 2. Materials and Methods

### 2.1. Materials

The composites of P(3HB-*co*-4HB) as polymer matrix and wood flour (WF) as natural filler were prepared using a micro-extruder MiniLab II (Thermo-Haake, Austin, TX, USA) equipped with co-rotating twin-screws. The rate of the screw rotating was 100 rpm. The bone shapes/dumbbell-shaped specimens were formed in MiniJet II (Thermo-Haake, Austin, TX, USA) mini-injection molding machine with a mold temperature of 60 °C. The samples were fabricated by injection molding (specimen type 1BA according to ISO 527-2:2012, [32] and type 1 according toISO 178:2019 [33]) with the processing parameters presented in Table 1. As polymer matrix and natural filler were using the commercially available powder P(3HB-*co*-4HB) with a number-average molar mass of *M_w_* = 625,000 g/mol and a molar mass dispersity of *M_w_/M_n_* = 2.5 with 8% of 4-hydroxybutyrate (4-HB) units (calculated by proton nuclear magnetic resonance spectroscopy) from Tianjin GreenBio Materials (Tianjin GuoYun Biological Material Co. Ltd., Tianjin, China) under the trade name: Sogreen 000a, and wood flour under the trade name Jeluxyl WEHO 500S from JELU-WERK (Rosenberg, Germany) supplied by Brenntag (Essen, Germany), respectively. Before compounding wood flour (obtained from pine tree and fir tree, distribution of particle size (sieve analysis): 46% < 180 µm, 96% < 75 µm; average width of particle: 25 µm; bulk density 180 kg/m^3^) was dried 4 h at 80 °C in a universal oven UF55 (Memmert GmbH + Co. KG, Schwabach, Germany). The dried wood flour was kept in a vacuum oven VO500 (Memmert GmbH + Co. KG, Schwabach, Germany). The following nisins from *Lactococcus lactis* were used: A-Nisin-1 from Sigma Aldrich (St. Louis, MO, USA) and B-Nisin-2 from Novazym Poland (Wielkopolska Centre of Advanced Technologies, Poznań, Poland).

### 2.2. Degradation Environments

#### 2.2.1. Aging Test under Laboratory Composting Conditions

The test was performed using Columbus Instruments S/N 110315 (Columbus, OH, USA) respirometer Micro-Oxymax equipped with a computer as a controller and a device for recording, archiving and presenting data. Micro-Oxymax performs periodic measurements in a closed system. The system, works under standard conditions, automatically compensates for changes in pressure and temperature [34]. For the test the samples were placed in glass jars containing 500 g of mature compost at a humidity level of 50% and then incubated for 3 weeks in an average temperature of 58 °C. For the biodegradation studies the composites were used in the form of dumbbell-shaped samples.

#### 2.2.2. Biodegradation Test under Industrial Composting Conditions

The biodegradation process was conducted using a BIODEGMA system [29]. For the test samples were placed in compost and then incubated for 3 weeks in an average temperature 63 °C under industrial conditions. After the incubation time, all samples were removed from compost, cleaned and analyzed [25].

#### 2.2.3. Abiotic Degradation

For the abiotic degradation experiments, samples were incubated in screw-capped vials with air-tight PTFE/silicone septa, containing 25 mL distilled water or phosphate buffer pH 7.4. The degradation experiment was conducted at 70 °C (± 0.5 °C) according to ISO 15814:1999 [35] as described elsewhere [26]. After a defined degradation time, the samples were separated from the degradation medium and dried under a vacuum at room temperature.

#### 2.2.4. Testing for Antimicrobial Activity

The antimicrobial activity of P(3HB-*co*-4HB) and P(3HB-*co*-4HB)/20WF with 5 wt. % of B-Nisin-2 and P(3HB-*co*-4HB) with B-Nisin-2 coating were investigated against the Gram-positive strain *Staphylococcus aureus* (NCIMB 6571) using the disc diffusion assay. Microbial strain was obtained from the University of Wolverhampton culture collection. Organisms were maintained at –20 °C in a lyophilized form. Stock culture of *S. aureus* was resuscitated on sterile tryptone soy agar (TSA) (Sigma-Aldrich, Saint Louis, MO, USA) and incubated for 48 h at 37 °C. Overnight broth cultures were aseptically prepared using the stock plates prior to experimental use. Discs with neat nisin (A-Nisin-1 and B-Nisin-2), P(3HB-*co*-4HB) with B-Nisin-2 coating or P(3HB-*co*-4HB) and P(3HB-*co*-4HB)/20WF with 5 wt. % of B-Nisin-2 were aseptically placed on TSA plates spread with overnight microbial cultures following incubation at 37 °C for 24 h, the zone of inhibitions (ZOI) were measured. Results were presented for ZOI (mm) at 24 h.

### 2.3. Characterization of the Samples

#### 2.3.1. Visual Examination

The surface of the materials was examined using scanning electron microscopy (SEM). SEM studies were performed by the Quanta 250 FEG (FEI Company, Fremont, CA, USA) high-resolution environmental scanning electron microscope operated at 10 kV acceleration voltage. The samples were observed without coating, under a low vacuum (80 Pa).

#### 2.3.2. Thermal Properties

Thermal characteristics of the samples were determined using the TGA/DSC1 Mettler-Toledo (Columbus, OH, USA) thermal analyzer from room temperature to 800 °C at a heating rate of 10 °C/min in a stream of nitrogen (60 mL/min). The differential scanning calorimetry (DSC) studies was done by means of TA DSC Q2000 apparatus (TA Instruments, New Castle, DE, USA). The instrument was calibrated with high purity indium. The first heating run in which the sample thermal history was suppressed was acquired from –80 to 200 °C at a heating rate of 20 °C/min. The second heating run from –80 to 200 °C (at a heating rate of 20 °C/min) was done for samples after rapid cooling from melt. The sample, after rapid cooling from 200 °C, was amorphous and allowed for finding the glass transition temperature. All of the experiments were performed under a nitrogen atmosphere, with a nitrogen flow rate of 50 mL/min.

#### 2.3.3. Water Uptake Measurement

Water uptake capacity X of the composite was measured after different times of incubation in water and buffer. From the difference of masses before and after immersion in water, the percentage of water uptake was calculated. The calculation was based on Equation (1) [36].

(1) $$X=\frac{m_{w}-m_{d}}{m_{d}}\;100\;\left(\%\right)$$

where *m_w_* and *m_d_* are the masses of the wet and dry samples, respectively.

#### 2.3.4. Multistage Electrospray Ionization Mass Spectrometry

Mass spectra were obtained from the Finnigan LCQ ion trap mass spectrometer (Finnigan, San Jose, CA, USA) operated in positive-ion mode. The degradation medium after degradation studied were lyophilized for 2 days. Then, the residue was dissolved in methanol/water system (1:1 vol.%). The solutions were introduced to the ESI source by continuous infusion using the instrument syringe pump at a flow rate of 5 µL/min. The ESI source of the LCQ was operating at 4.50 kV and the capillary heater was set to 200 °C. Nitrogen was used as the sheath gas, helium was used as the auxiliary gas. For ESI–MS^n^ experiments, monoisotopic ions of interest were isolated in the ion trap and activated using helium damping gas in the mass analyzer to promote collisions (CID).

#### 2.3.5. Mechanical Properties

The mechanical properties of wood flour-based composites were determined by tensile tests and flexural tests (three-point bending tests). Tests were performed using Instron 4204 universal testing machine (Norwood, MA, USA) equipped with a 1 kN cell load. Preload of 0.010 kg for tests were used. Tensile tests were carried out according to standard ISO 527-2:2012 [32]. The crosshead speed was 20 mm/min. Flexural tests were carried out according to standard ISO 178:2019 [33]. The crosshead speed was 2 mm/min. The system controls and data analysis were performed using the supplied Instron IX.

## 3. Results and Discussion

### 3.1. Mechanical Properties

The influence of used filler and filler content on mechanical properties of composites were examined in tensile mode and flexural mode (three-point bending). The results of mechanical tests realized in tensile are summarized in Table 2 for tensile strength, Young modulus and elongation at break.

Composites with wood flour as filler exhibit decreased tensile strength value vs. neat matrix correspondingly to filler content growth. Observed result is a consequence a weak adhesion between polymer matrix and filler. Presence of filler without coupling agent under processing exhibits reduced tensile strength for composites with increase filler content. Wood flour is responsible for discontinuity of polymer matrix at tensile load. The Young modulus values of composites with 10, 20, and 30 wt. % of filler are average of 979, 1326, and 1632 MPa, respectively. The neat P(3HB-*co*-4HB) reveal Young modulus value of 964 MPa. Composites with 20 and 30 wt. % of filler are more rigid than neat matrix. Composites with 10 wt. % of wood flour obtained very similar result, consider standard deviation value, to neat matrix. Findings can explain a form of filler. Wood flour at 10 wt. % content in composites did not have influence on stiffness of obtained composites in comparison with neat P(3HB-*co*-4HB). This behavior is consequence of low dispersion of wood flour in matrix. Wood flour used as filler in tested composites was obtained from coniferous trees (pine tree and fir tree), which are softer filler in comparison with wood flour receive from deciduous tree. Also, distribution of particle size of 96% < 75 µm and average width of particle of 25 µm, are also factors which exhibit influence on mechanical properties correspondingly to filler content in composites. These reasons decide that suitable/adequate wood flour content exhibit influence on stiffness [37,38,39]. Results of mechanical tests at flexural mode are summarized in Table 3.

Flexural strength is lower for composites than for neat matrix. Reduction of the flexural strength is more significant with the increase of filler content. The observed response is connected with reduced elasticity of composites. Wood flour decreased P(3HB-*co*-4HB) matrix ability to bending stress transfer. The value of flexural modulus increased with increasing of filler content. Composites are stiffer than neat matrix. Standard deviation values of the results of composites with 30 wt. % of filler is high (258 MPa) and is connected with non-uniform filler distribution in composite specimens, which is a consequence of the formation filler aggregates.

### 3.2. Macroscopic Changes

**Figure 1:**
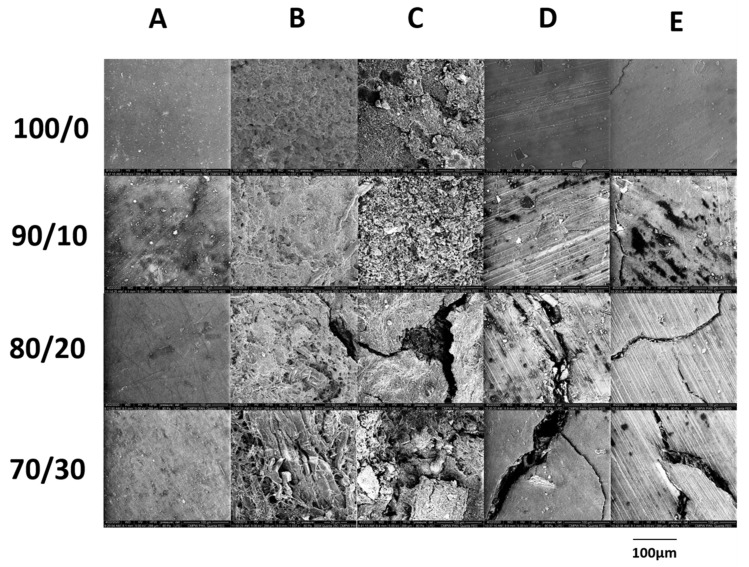
SEM micrographs of the neat P(3HB-*co*-4HB) (100/0) and P(3HB-*co*-4HB)/WF composites with the mass ratio of 90/10, 80/20, and 70/30, before (**A**) and after 21 days of degradation in BIODEGMA (**B**), Respirometer (**C**), water (**D**), and buffer (**E**). (Original SEM photos in the Supplementary Material Figure S1).

Comparing the SEM images of samples incubated 21 days in different environments the variances in their structure could be noticed (Figure 1). For samples degraded under abiotic conditions, the pinholes formation was observed, indicating water penetration and degradation in the deeper parts of the samples. Increasing of the filler amount in the composites cause the increasing in the erosion of the samples surface after degradation in water and buffer. Natural wood flour is a porous and hydrophilic material, the weak interaction between hydrophilic filler and hydrophobic polymer matrix could create a new path for water penetration, which results in increased hydrolytic degradation of the tested samples and formation of pinholes. During degradation under composting conditions larger changes were observed on the surface of the samples, suggesting the dominance of enzymatic degradation. It can be seen that in these conditions, changes of the sample surfaces do not depend on filler amount. However, pinholes that indicate water penetration have also been observed. Thus, from a macroscopic observation, it can be concluded that the mechanisms of both hydrolytic and enzymatic degradation are possible during the incubation in compost of the samples tested.

### 3.3. Thermal Behavior

**Figure 2:**
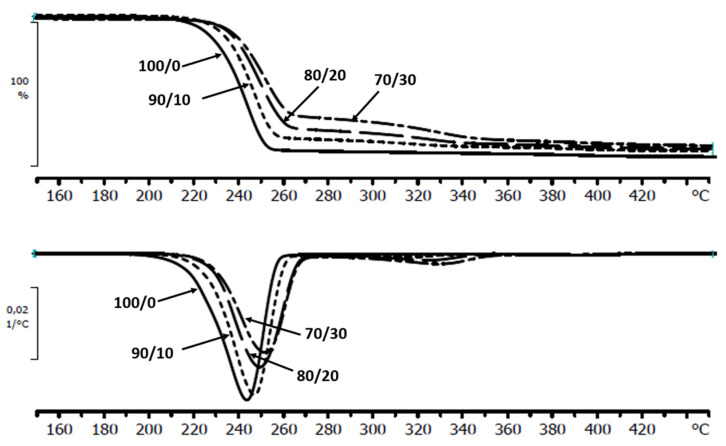
Thermal decomposition (TG) curves and their first-order derivative (DTG) curves of the neat P(3HB-*co*-4HB) (100/0) and P(3HB-*co*-4HB)/WF composites with the mass ratio of 90/10, 80/20, and 70/30, before degradation.

The thermal decomposition curves of the neat matrix before and after degradation in the investigated environments followed a single mass-loss step. For the composites, the second step of mass loss occurred. The first step of mass loss refers to the thermal degradation of polymer matrix, the second to the thermal degradation of wood components. The addition of the natural filler caused the increase of the temperature of the maximum decomposition rate (*T_max_*) of obtained materials. The stability of composites increases with the increase of wood flour (Figure 2).

After degradation, a further increase in the thermal stability of the polymer matrix was observed for all investigated samples (Table 4.).

**Figure 3:**
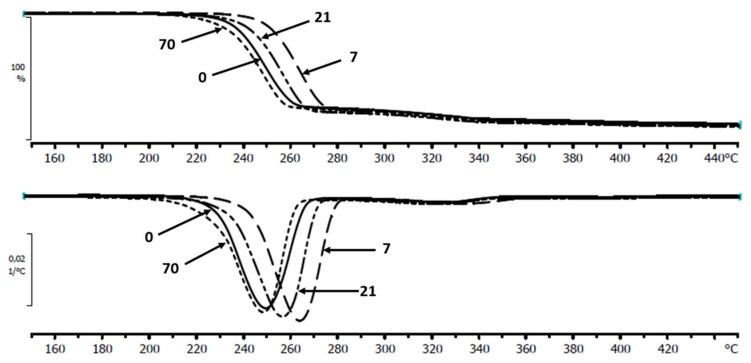
Selected TG and DTG thermograms of the P(3HB-*co*-4HB)/20WF composite before and after specific time (days) of incubation in buffer.

The factor affecting this phenomenon may be the presence of the acidic low-molar-mass degradation products or acidic components released from wood flour, similarly as it was observed in composites containing cork [29]. This conclusion was indirectly confirmed by the results obtained for the composites during degradation in a slightly alkaline buffer (pH = 7.4) where during incubation the *T_max_* starts to decrease after 7 days of incubation for a P(3HB-*co*-4HB)/WF composites (Figure 3).

**Figure 4:**
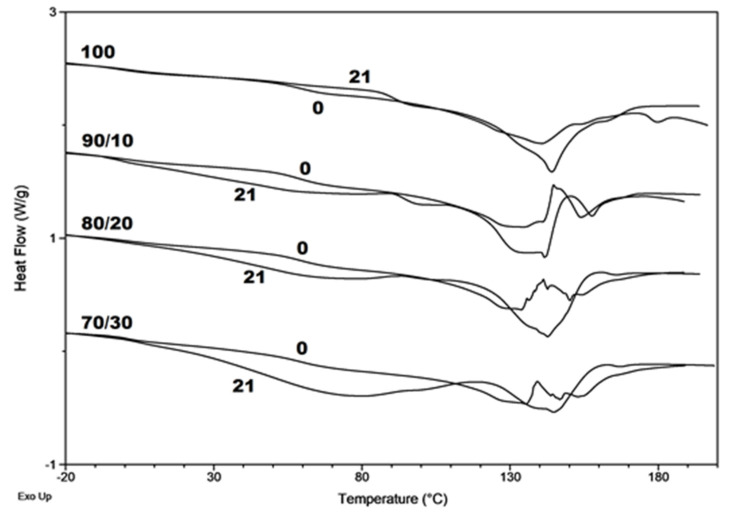
Selected differential scanning calorimetry (DSC) curves (first heating run) for the neat P(3HB-*co*-4HB) (100/0) and P(3HB-*co*-4HB)/WF composites with the mass ratio of 90/10, 80/20, and 70/30, before (0) and after 21 days degradation in water (21).

The DSC traces exhibited multiple melting endotherms for investigated sample before degradation. This phenomenon has been also reported for many semi-crystalline polymers [18,40]. It is apparent from the DSC traces of neat P(3HB-*co*-4HB) (100/0) that after 21 days of incubation in water a sharper melting peak was observed. This suggests that after degradation in these conditions, more regular crystals were obtained. For the composites this phenomenon is not so clear (Figure 4).

DSC results of the first and the second heating run for neat polymer matrix and composites are listed in Table 5. The addition of wood flour caused the increase of glass transition temperature (*T_g_*) value, indicating the appearance of physical entanglement, and the increase of melting enthalpy *(*Δ*H_m_*). It seems that the wood flour initiates crystallization as a nucleation agent.

After degradation of neat P(3HB-*co*-4HB) matrix in all investigated environments, an increase in Δ*H_m_* was observed. The behavior was different for composites. In the composting conditions for composites with less filler content (10 and 20 wt. %), an increase in Δ*H_m_* was observed, while for a composite with a content of 30 wt. % decrease. For example, after 21 days of degradation in BIODEGMA of the P(3HB-*co*-4HB)/30WF sample the Δ*H_m_* decrease to 66.5 J/g. After 21 days of incubation in water the decrease of Δ*H_m_* was observed also for P(3HB-*co*-4HB)/20WF and after degradation in buffer this phenomenon was observed for all composites. For these types of materials, both the nucleation effect of the degradation products and the wood flour itself, as well as the degradation mechanism, can affect the degree of crystallization. The higher amount of the wood flour studied reduced the additional mobility of the polymer chains, which caused in crystallinity decrease [41]. At the same time, a larger degradation of the polymer matrix in this composite may occur, which may also cause a change in crystallinity.

### 3.4. Abiotic Degradation

#### 3.4.1. Material Examination

**Figure 5:**
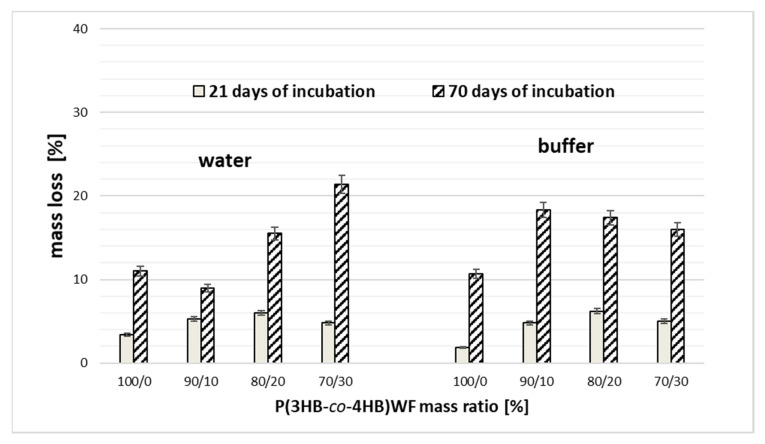
Mass loss of neat P(3HB-*co*-4HB) and P(3HB-*co*-4HB)/WF composites after 21 and 70 days of degradation.

**Figure 6:**
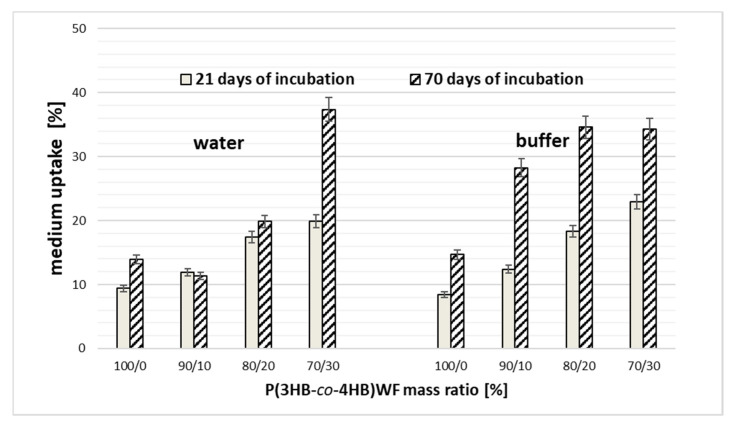
Medium uptake after 21 and 70 days of degradation of neat P(3HB-*co*-4HB) and P(3HB-*co*-4HB)/WF composites.

Evaluation of mass changes of the specimens’ during degradation in compost is very complicated due to the cleaning process from a compost environment, especially after disintegration of the specimens had taken place. Thus, Figure 5 presents the mass loss only for samples after degradation in water and buffer. The determined value of the sample’s mass loss, after 21 days of incubation in both water and buffer, increasing with the increase of the wood flour content till 20 wt. % in the composites studied. After degradation in water the significant effect of the wood flour content on the mass loss was found after 70 days of degradation which can be result from improving water penetration into the disintegrated samples. This phenomenon can be explained by the hydrophilic character of the filler and water uptake. Wood flour absorbed water, in proportion to its content in the composites, from the beginning of the incubation process (Figure 6). A similar observation has been reported in the studies on PLA degradation [42]. In the case of P(3HB-*co*-4HB)/10WF composite, the reverse trend is observed. Ten weight percent of the filler can easily be entrapped within the polymer matrix, which resulted in less water uptake by the composite and lower mass loss after 70 days of incubation (Figure 5 and Figure 6). Degradation in a buffer at a constant pH leads to decrease of mass loss after 70 days of incubation with the filler amount increase (Figure 5) which can be resulted by the reduction of autocatalytic effect. This could be caused by the better penetration of samples by the buffer.

In composites containing the wood flour above 20 wt. %, the advantageous influence of the filler on the degradation process was found. The increase in uptake of the medium for composites correlated with the increase in their mass loss due to sample hydrolysis as compared with neat P(3HB-*co*-4HB). Samples containing 30 wt. % of wood flour had the highest, more than 20%, mass loss and water uptake on a level above 35% after 70 days incubation in water (Figure 6). This behavior is opposite to the previously observed slowing degradation effect of the cork as a filler in composites [29].

#### 3.4.2. Analysis of Degradation Products

**Figure 7:**
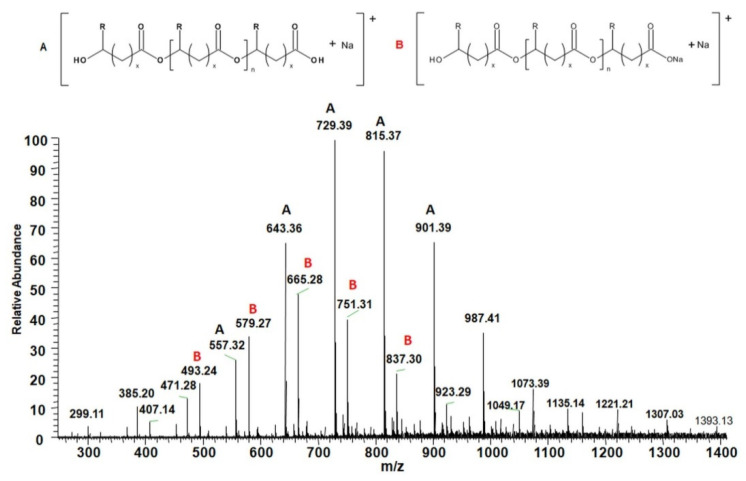
ESI–MS spectra (in positive ion mode) of oligo(3HB-*co*-4HB)/30WF after 70 days of degradation in water (for 3-HB units R = CH_3_ and x = 1; for 4-HB units R = H and x = 2).

Recently, the multistage electrospray ionization mass spectrometry technique is an irreplaceable tool for the structural characterization of (bio)degradable polymers. This method provides valuable information about the monomer units and their sequences in the polymer chain [43]. The molecular structure of obtained degradation products of P(3HB-*co*-4HB) released to the water were verified by using the ESI–MS^n^. Figure 7 shows the positive-ion spectra of oligomers of P(3HB-*co*-4HB) after 70 days of incubation in water. The spectra consist of two series of sodium charged ions with different degrees of oligomerization. However, the monomer units of 3-hydroxybutyrate (3-HB) and 4-hydroxybutyrate (4-HB) are indistinguishable by mass spectra because they have the same molar mass (86 Da) [44]. The main series of ions on the spectra (labeled as series A) that appears at *m/z* = [104 + (86·n)] + 23 (n is a number of HB repeating units) correspond to the sodium adducts of oligo(3-hydroxybutyrate-*co*-4-hydroxybutyrate) (oligo(3HB-*co*-4HB)) chains terminated by carboxyl and hydroxyl end groups. Whereas, the second series of ions (labeled as series B) correspond to the sodium adducts of oligo(3-hydroxybutyrate-*co*-4-hydroxybutyrate) sodium salt of chains terminated by carboxylate and hydroxyl end groups.

**Figure 8:**
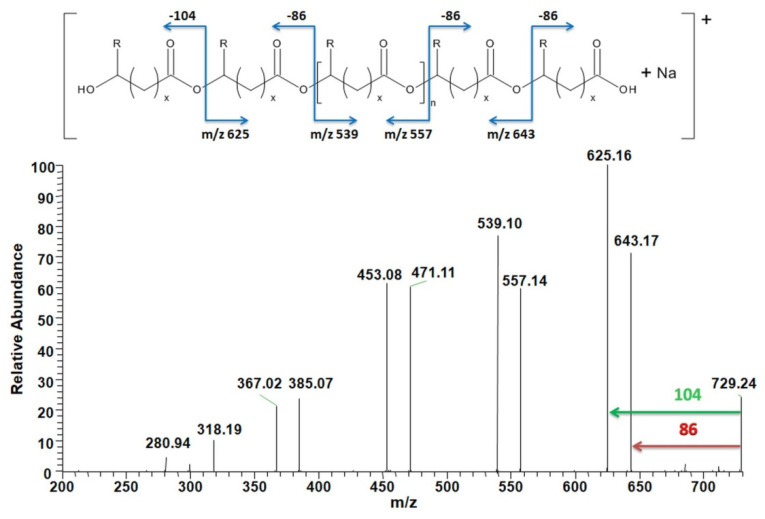
ESI–MS/MS fragmentation experiment for the ion at *m/z* 729 of oligo(3HB-*co*-4HB)/30WF after 70 days of degradation in water (for 3-HB units R = CH_3_ and x = 1; for 4-HB units R = H and x = 2) (see Figure 7).

To confirm the chemical structures of the degradation products ESI–MS/MS was applied. Figure 8 shows the ESI–MS/MS spectra obtained for the ion at *m/z* 729, which corresponds to the sodium adduct oligo(3HB-*co*-4HB) terminated by hydroxyl and carboxyl end group. According to the structure assigned, the product ion at *m/z* 625 corresponds to the oligomers formed by the expulsion of 3-hydroxybutyric acid (104 Da), and the product ion at *m/z* 643 corresponds to the oligomers formed by the loss of a neutral molecule of crotonic acid (86 Da) [45]. For all the tested samples, the same degradation products were determined.

### 3.5. Testing for Antimicrobial Activity

The feature of the active packaging, such as extending food shelf life, can be achieved by introducing the antimicrobial agents into materials, which (after releasing) inhibit or retard microbial growth on food surfaces. A very interesting application of composites with antimicrobial agents can be disposable tableware. The bioactivity seems to be an additional advantage for this type of application as it can limit the spared of pathogenic microbes such as *S. aureus* in the hospital environment. Initially antimicrobial activity was tested for neat nisin (A-Nisin-1 and B-Nisin-2) as well as the P(3HB-*co*-4HB) and P(3HB-*co*-4HB)/20WF with 5 wt. % of B-Nisin-2 against Gram-positive *S. aureus*. The clear zones of inhibition were observed only on the plates with neat nisin (A-Nisin-1 and B-Nisin-2) (Table 6). This preliminary research indicated a possibility of application of nisin as an additive to biodegradable composites to extend their use. Following the initial findings the P(3HB-*co*-4HB) with B-Nisin-2 coating was tested for microbial activity.

The obtained results after 24 h of incubation of the P(3HB-*co*-4HB) and P(3HB-*co*-4HB)/20WF with 5 wt. % of B-Nisin-2 resulted in lack of antimicrobial activity therefore the observations suggest that during processing of the composite, the antimicrobial agent is either entrapped within the polymer matrix or inactivated during the preparation process. On the other hand, applying a nisin coating on the surface of the sample allowed to obtain a material showing antimicrobial activity.

## 4. Conclusions

In this paper, the (bio)degradation under laboratory and industrial composting conditions of composites made of P(3HB-*co*-4HB) and wood flour as well as the antimicrobial activity of selected materials with nisin were studied. The accelerating of the abiotic degradation process via addition of wood flour has been shown. For the composites obtained from P(3HB-*co*-4HB) a significant influence of wood flour presence on thermal properties changes of tested materials during degradation was found. The addition of filler affected on their mechanical properties, especially the stiffness of composites was higher than for neat matrix. The acidic products from hydrolytic degradation and the compounds derived from filler presented in the studied samples were responsible for increasing of their thermal stability. The P(3HB-*co*-4HB) composites with wood flour were proposed for food-packaging applications. The applying a nisin coating on the surface of the selected material can extends the protective capacity of such packaging.

## Figures and Tables

**Table 1:** Processing parameters of composites.

P(3HB-*co*-4HB)/WF (Mass Ratio)	Temperature of Plasticizing Zone (°C)	Injection Temperature (°C)	Injection Pressure (bar)
100	140	140	350
90/10	140	140	450
80/20	140	140	550
70/30	150	150	700
(100)/5 wt. % of nisin	140	140	350
(80/20)/5 wt. % of nisin	140	140	550

**Table 2:** Tensile properties of P(3HB-*co*-4HB) composites containing the wood flour.

P(3HB-*co*-4HB)/WF (Mass Ratio)	Tensile Strength σ_m_		Young Modulus Ε_t_		Elongation at Break ε_b_	
	Mean (MPa)	Sd (MPa)	Mean (MPa)	Sd (MPa)	Mean (%)	Sd (%)
100/0	24.9	1.0	964	29	8.5	1.0
90/10	22.2	0.3	974	38	7.1	1.1
80/20	21.8	1.2	1326	56	4.1	0.3
70/30	20.7	1.1	1632	48	3.7	0.5

Sd—standard deviation.

**Table 3:** Result of flexural strength and modulus of elasticity in flexure determined under three-point bending tests of P(3HB-co-4HB) composites with wood flour.

P(3HB-*co*-4HB)/WF (Mass Ratio)	Flexural Strength σ_fM_		Modulus of Elasticity in Flexure Ε_f_	
	Mean (MPa)	Sd (MPa)	Mean (MPa)	Sd (MPa)
100/0	38.6	1.0	1169	55
90/10	35.1	1.4	1412	96
70/30	28.2	1.8	1791	258

Sd—standard deviation.

**Table 4:** Thermogravimetric parameters before and after 21 days of degradation of neat P(3HB-*co*-4HB) and P(3HB-*co*-4HB)/WF composites.

P(3HB-*co*-4HB)/WF (Mass Ratio)	Environment	*T_max_* (°C)
100/0	Before degradation	243
90/10		247/316
80/20		249/324
70/30		251/329
100/0	BIODEGMA	258
90/10		268/336
80/20		272/339
70/30		270/333
100/0	Respirometer	263
90/10		283/360
80/20		279/359
70/30		287/366

**Table 5:** Calorimetric parameters of neat P(3HB-*co*-4HB) and P(3HB-*co*-4HB)/WF composites before and after 21 days of degradation in different environments, 20 °C/min; *T_m_*—melting temperature and ∆*H_m_*—melting enthalpy (first heating scan), *T_g_* (second heating scan after rapid cooling) (thermograms in the Supplementary Material Figure S2).

P(3HB-*co*-4HB)/WF (Mass Ratio)	Environment	*T_g_* (°C)	*T_m_* (°C)	Δ*H_m_* (J/g)
100/0	Before degradation	−8.7	140.7/179.4	21.0
90/10		−5.9	141.2/162.8	32.2
80/20		−3.7	139.3/158.7	48.2
70/30		−1.4	140.7/154.9	81.2
100/0	BIODEGMA	−4.2	147.0	42.1
90/10		−1.9	142.5/161.4	43.6
80/20		1.0	102.6/140.1/154.5	61.1
70/30		0.4	99.0/140.0/153.3	66.5
100/0	Respirometer	1.8	146.3/164.3	37.4
90/10		0.3	144.1/163.5	50.2
80/20		−0.1	82.8/137.1/149.7	57.8
70/30		0.4	82.2/135.2/151.5	79.0
100/0	water	−1.6	144.1	43.8
90/10		−3.1	141.6/157.4	47.7
80/20		−3.8	142.7/165.5	47.3
70/30		−2.3	77.5/144.4/167.2	77.4
100/0	buffer	−3.0	144.7	36.4
90/10		−6.2	141.9/158.4	29.7
80/20		−3.6	145.2	46.2
70/30		−6.6	87.6/150.4	63.7

**Table 6:** Antimicrobial activity against *Staphylococcus* *aureus* (NCIMB 6571) using the disc diffusion assay.

Sample	*Staphylococcus aureus* ZOI (mm)
A-Nisin-1	5.0 ± 1
B-Nisin-2	7.0 ± 1
P(3HB-*co*-4HB) with 5 wt. % of B-Nisin-2	not detected
P(3HB-*co*-4HB)/20WF with 5 wt. % of B-Nisin-2	not detected
P(3HB-*co*-4HB) coated with B-Nisin-2	3.5 ± 1

## Supplementary Materials

The following are available online at https://www.mdpi.com/1996-1944/13/9/2200/s1, Figure S1: SEM micrographs, Figure S2: DSC plot.

## References

[1] Koller M. (2014). Poly(hydroxyalkanoates) for food packaging: Application and attempts towards implementation. Appl. Food Biotechnol..

[2] Ahmeda J., Varshney S.K. (2011). Polylactides—Chemistry, properties and green packaging technology: A review. Int. J. Food Prop..

[3] Musioł M., Janeczek H., Jurczyk S., Kwiecień I., Sobota M., Marcinkowski A., Rydz J. (2015). (Bio) degradation studies of degradable polymer composites with jute in different environments. Fibers Polym..

[4] Barkoula N.M., Garkhail S.K., Peijs T. (2010). Biodegradable composites based on flax/polyhydroxybutyrate and its copolymer with hydroxyvalerate. Ind. Crops Prod..

[5] Javadi A., Srithep Y., Pilla S., Lee J., Gong S., Turng L.-S. (2010). Processing and characterization of solid and microcellular PHBV/coir fiber composites. Mater. Sci. Eng. C.

[6] Liu R., Cao J., Ou-Yang L. (2013). Degradation of wood flour/poly(lactic acid) composites reinforced by coupling agents and organo-montmorillonite in a compost test. Wood Fiber.

[7] Daian G., Ozarska B. (2009). Wood waste management practices and strategies to increase sustainability standards in the Australian wooden furniture manufacturing sector. J. Clean. Prod..

[8] Directive (EU) 2019/904 of the European Parliament and of the Council of 5 June 2019 on the Reduction of the Impact of Certain Plastic Products on the Environment. https://eur-lex.europa.eu/eli/dir/2019/904/oj.

[9] Fernandes E.G., Pietrini M., Chiellini E. (2004). Bio-based polymeric composites comprising wood Flour as filler. Biomacromolecules.

[10] Luo S., Netravali A.N. (1999). Interfacial and mechanical properties of environment-friendly “green” composites made from pineapple fibers and poly(hydroxybutyrate-*co*-valerate) resin. J. Mater. Sci..

[11] Teymoorzadeh H., Rodrigue D. (2015). Biocomposites of wood flour and polylactic acid: Processing and properties. J. Biobased Mater. Bioenergy.

[12] Zhang X., Wu X., Haryono H., Xia K. (2014). Natural polymer biocomposites produced from processing raw wood Flour by severe shear deformation. Carbohydr. Polym..

[13] Rodríguez F.J., Torres A., Peñaloza Á., Sepúlveda H., Galotto M.J., Guarda A., Bruna J. (2014). Development of an antimicrobial material based on a nanocomposite cellulose acetate film for active food packaging. Food Addit. Contam. Part A.

[14] Sirviö J.A., Liimatainen H., Niinimäki J., Hormi O. (2013). Sustainable packaging materials based on wood cellulose. RSC Adv..

[15] Laxmeshwar S.S., Kumar D.J.M., Viveka S., Nagaraja G.K. (2012). Preparation and properties of biodegradable film composites using modified cellulose fibre-reinforced with PVA. Polym. Sci..

[16] Vandi L.-J., Chan C.M., Werker A., Richardson D., Laycock B., Pratt S. (2018). Wood-PHA composites: Mapping opportunities. Polymers.

[17] Swift G. (2015). Degradable polymers and plastics in landfill sites. Encyclopedia of Polymer Science and Technology.

[18] Musioł M., Sikorska W., Adamus G., Janeczek H., Kowalczuk M., Rydz J. (2015). (Bio)degradable polymers as a potential material for food packaging: Studies on the(bio)degradation process of PLA/(R,S)-PHB rigid foils under industrial composting conditions. Eur. Food Res. Technol..

[19] Sikorska W., Musiol M., Nowak B., Pajak J., Labuzek S., Kowalczuk M., Adamus G. (2015). Degradability of polylactide and its blend with poly [(R,S)-3-hydroxybutyrate] in industrial composting and compost extract. Int. Biodeterior. Biodegrad..

[20] Rydz J., Sikorska W., Kyulavska M., Christova D. (2015). Polyester-based (bio)degradable polymers as environmentally friendly materials for sustainable development. Int. J. Mol. Sci..

[21] Musioł M., Sikorska W., Janeczek H., Wałach W., Hercog A., Johnston B., Rydz J. (2018). (Bio)degradable polymeric materials for a sustainable future—Part 1: Organic recycling of PLA/PBAT blends in the form of prototype packages with long shelf-life. Waste Manag..

[22] Polyaka P., Szemerszkia D., Voros G., Pukanszky B. (2017). Mechanism and kinetics of the hydrolytic degradation of amorphous poly(3-hydroxybutyrate). Polym. Degrad. Stab..

[23] Musioł M., Sikorska W., Adamus G., Kowalczuk M. (2016). Forensic engineering of advanced polymeric materials. Part III—Biodegradation of thermoformed rigid PLA packaging under industrial composting conditions. Waste Manag..

[24] Rydz J., Wolna-Stypka K., Adamus G., Janeczek H., Musioł M., Sobota M., Marcinkowski A., Krzan A., Kowalczuk M. (2015). Forensic engineering of advanced polymeric materials. Part 1—Degradation studies of polylactide blends with atactic poly[(R,S)-3-hydroxybutyrate] in paraffin. Chem. Biochem. Eng. Q..

[25] Sikorska W., Rydz J., Wolna-Stypka K., Musioł M., Adamus G., Kwiecień I., Janeczek H., Duale K., Kowalczuk M. (2017). Forensic engineering of advanced polymeric materials—Part V: Prediction studies of aliphatic-aromatic copolyester and polylactide commercial blends in view of potential applications as compostable cosmetic packages. Polymers.

[26] Sikorska W., Richert J., Rydz J., Musioł M., Adamus G., Janeczek H., Kowalczuk M. (2012). Degradability studies of poly(L-lactide) after multi-reprocessing experiments in extruder. Polym. Degrad. Stab..

[27] Gonzalez Ausejo J., Rydz J., Musioł M., Sikorska W., Sobota M., Włodarczyk J., Adamus G., Janeczek H., Kwiecień I., Hercog A. (2018). A comparative study of three-dimensional printing directions: The degradation and toxicological profile of a PLA/PHA blend. Polym. Degrad. Stab..

[28] Gonzalez Ausejo J., Rydz J., Musioł M., Sikorska W., Janeczek H., Sobota M., Włodarczyk J., Szeluga U., Hercog A., Kowalczuk M. (2018). Three-dimensional printing of PLA and PLA/PHA dumbbell-shaped specimens of crisscross and transverse patterns as promising materials in emerging application areas: Prediction study. Polym. Degrad. Stab..

[29] Jurczyk S., Musioł M., Sobota M., Klim M., Hercog A., Kurcok P., Janeczek H., Rydz J. (2019). (Bio)degradable polymeric materials for sustainable future—Part 2: Degradation studies of polymer-cork composites in different environments. Polymers.

[30] Meira S.M.M., Zehetmeyer G., Jardim Z.A., Scheibel J.M., Vinicius R., de Oliveira R.V.B., Brandelli A. (2014). Polypropylene/montmorillonite nanocomposites containing nisin as antimicrobial food packaging. Food Bioprocess Technol..

[31] Shiroodi S.G., Nesaei S., Ovissipour M., Al-Qadiri H.M., Rasco B., Sablani S. (2016). Biodegradable polymeric films incorporated with nisin: Characterization and efficiency against *Listeria monocytogenes*. Food Bioprocess Technol..

[32] ISO 527-2:2012 (2012). Plastics—Determination of Tensile Properties—Part 2: Test Conditions for Moulding and Extrusion Plastics.

[33] ISO 178:2019 (2019). Plastics—Determination of Flexural Properties.

[34] Columbus Instruments. http://www.colinst.com.

[35] International Standard: ISO 15814 (1999). Implants for Surgery—Copolymers and Blends Based on Polylactide—In Vitro Degradation Testing.

[36] Zahari W.Z.W., Badri R.N.R.L., Ardyananta H., Kurniawan D., Nor F.M. (2015). Mechanical properties and water absorption behavior of polypropylene/Ijuk fiber composite by using silane treatment. Procedia Manuf..

[37] Farsi M. (2010). Wood-plastic composites: Influence of wood flour chemical modification on the mechanical performance. J. Reinf. Plast. Compos..

[38] Poletto M., Zattera A.J., Forte M.M.C., Santana R.M.C. (2012). Thermal decomposition of wood: Influence of wood components and cellulose crystallite size. Bioresour. Technol..

[39] Febrianto F., Setyawati D., Karina M., Bakar E.S., Hadi Y.S. (2006). Influence of wood flour and modifier contents on the physical and mechanical properties of wood Flour-recycle polypropylene composites. J. Biol. Sci..

[40] Wang Y., Mano J.F. (2005). Influence of melting conditions on the thermal behavior of poly(l-lactic acid). Eur. Polym. J..

[41] Han X., Pan J. (2009). A model for simultaneous crystallization and biodegradation of biodegradable polymers. Biomaterials.

[42] Petinakis E., Liu X., Yu L., Way C., Sangwan P., Dean K., Bateman S., Edward G. (2010). Biodegradation and thermal decomposition of poly(lactic acid)-based materials reinforced by hydrophilic fillers. Polym. Degrad. Stab..

[43] Johnston B., Radecka I., Chiellini E., Barsi D., IvanovaIlieva V., Sikorska W., Musioł M., Zięba M., Chaber P., Marek A.A. (2019). Mass spectrometry reveals molecular structure of polyhydroxyalkanoates attained by bioconversion of oxidized polypropylene waste fragments. Polymers.

[44] Kwiecień M., Kawalec M., Kurcok P., Kowalczuk M., Adamus G. (2014). Selective carboxylate induced thermal degradation of bacterial poly(3-hydroxybutyrate-*co*-4-hydroxybutyrate)—Source of linear uniform 3HB4HB oligomers. Polym. Degrad. Stab..

[45] Kwiecień I., Radecka I., Kowalczuk M., Adamus G. (2015). Transesterification of PHA to Oligomers Covalently Bonded with (Bio)Active Compounds Containing Either Carboxyl or Hydroxyl Functionalities. PLoS ONE.

