# Supplementary material for: (Bio)Degradable Polymeric Materials for Sustainable Future—Part 3: Degradation Studies of the PHA/Wood Flour-Based Composites and Preliminary Tests of Antimicrobial Activity
Source: Materials (Basel). 2020 May 11;13(9):2200. doi: 10.3390/ma13092200 (PMC7254317; doi:10.3390/ma13092200)
Supplement: Supplementary file 1 [file materials-13-02200-s001.pdf]

materials

Article

Supplementary Materials: (Bio)Degradable Polymeric Materials for Sustainable Future—Part 3: Degradation Studies of the PHA/Wood Flour-Based Composites and Preliminary Tests of Antimicrobial Activity

Marta Musioł [1,*], Sebastian Jurczyk [2], Michał Sobota [1], Magdalena Klim [1,3], Wanda Sikorska [1], Magdalena Zięba [1], Henryk Janeczek [1], Joanna Rydz [1], Piotr Kurcok [1], Brian Johnston [4], and Izabela Radecka [4]

[1] Centre of Polymer and Carbon Materials, Polish Academy of Sciences, 34. M. Curie-Sklodowska St., 41-819 Zabrze, Poland; msobota@cmpw-pan.edu.pl (M.S.); klim.magdalena@gmail.com (M.K.); wsikorska@cmpw-pan.edu.pl (W.S.); mzieba@cmpw-pan.edu.pl (M.Z.); hjaneczek@cmpw-pan.edu.pl (H.J.); jrydz@cmpw-pan.edu.pl (J.R.); pkurcok@cmpw-pan.edu.pl (P.K.);

[2] Łukasieiwcz Research Network – Institute for Engineering of Polymer Materials and Dyes, 55, M. Sklodowska-Curie St., 87-100 Toruń, Poland; s.jurczyk@impib.pl

[3] Department of Microbiology and Virology, School of Pharmacy with the Division of Laboratory Medicine, Medical University of Silesia, 4 Jagiellońska St., 41-200 Sosnowiec, Poland

[4] Wolverhampton School of Sciences, Faculty of Science and Engineering, University of Wolverhampton, Wulfruna Street, Wolverhampton, WV1 1LY, UK; B.Johnston@wlv.ac.uk (B.J.); I.Radecka@wlv.ac.uk (I.R.)

* Correspondence: mmusiol@cmpw-pan.edu.pl; Tel.: +48-322-716-077

Received: 15 April 2020; Accepted: 8 May 2020; Published: date

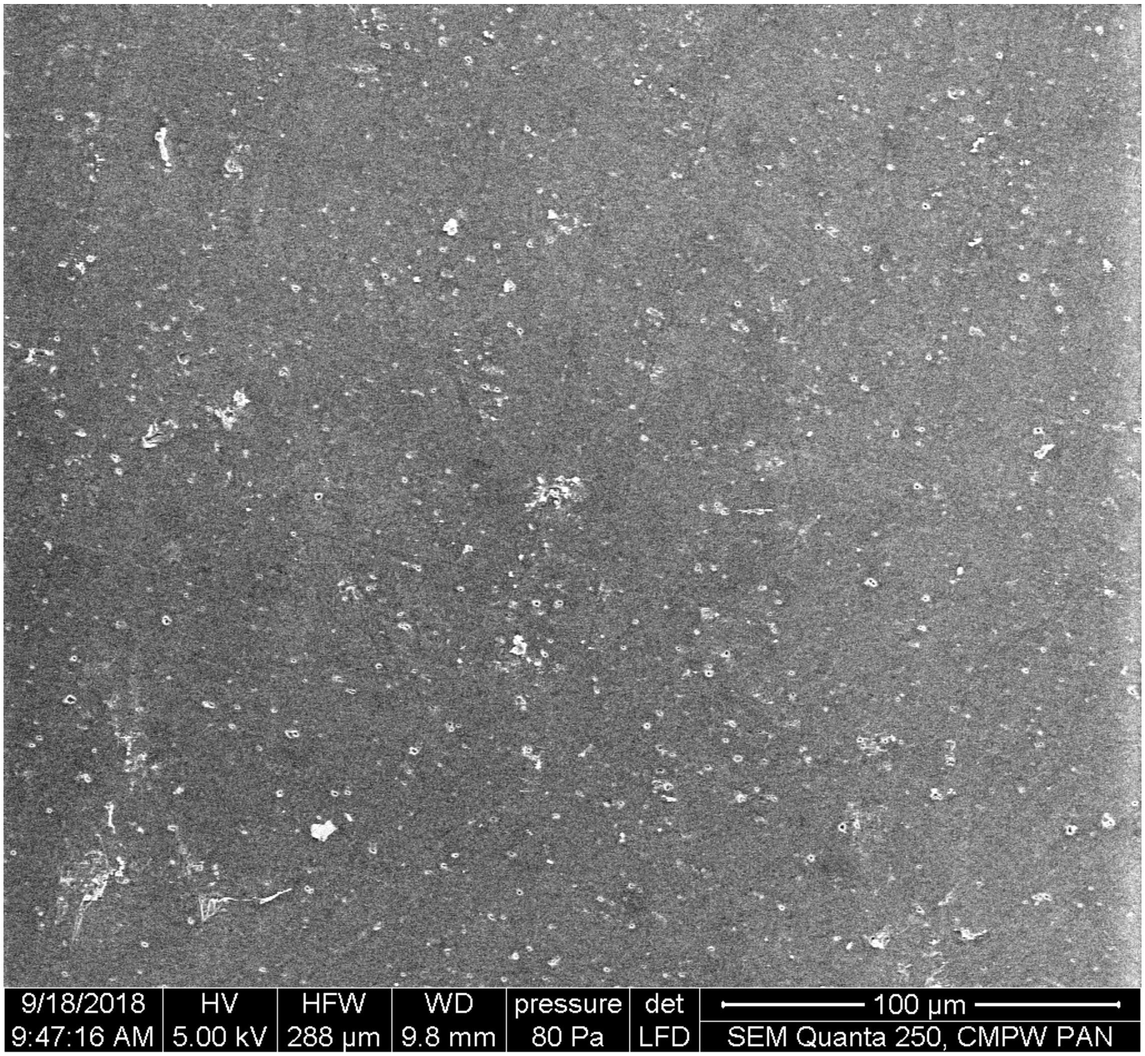

(**a**) SEM micrographs of the neat P(3HB-*co*-4HB) (100/0) before degradation.

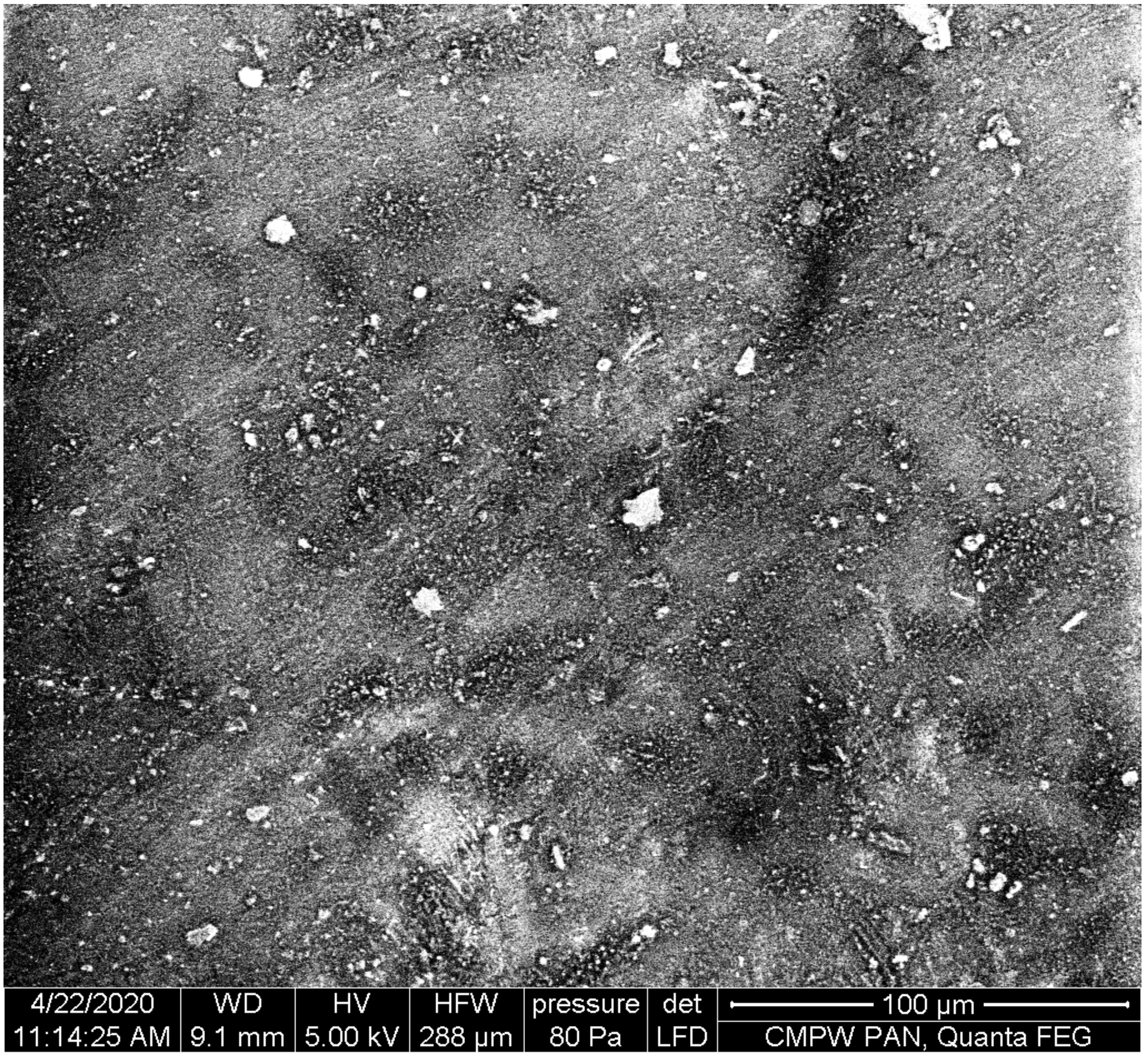

(**b**) SEM micrographs of the P(3HB-*co*-4HB)/10WF before degradation.

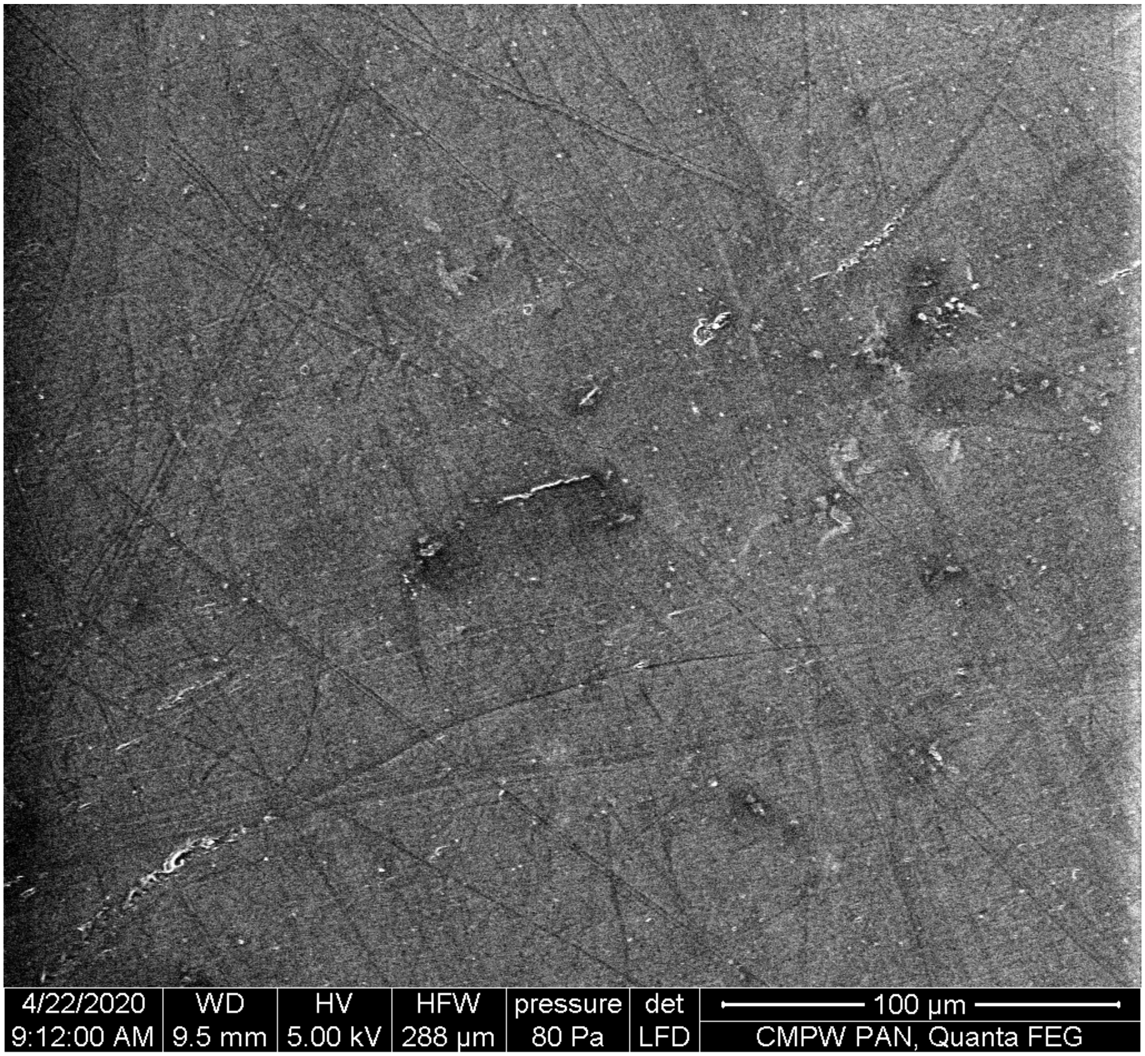

(**c**) SEM micrographs of the P(3HB-*co*-4HB)/20WF before degradation.

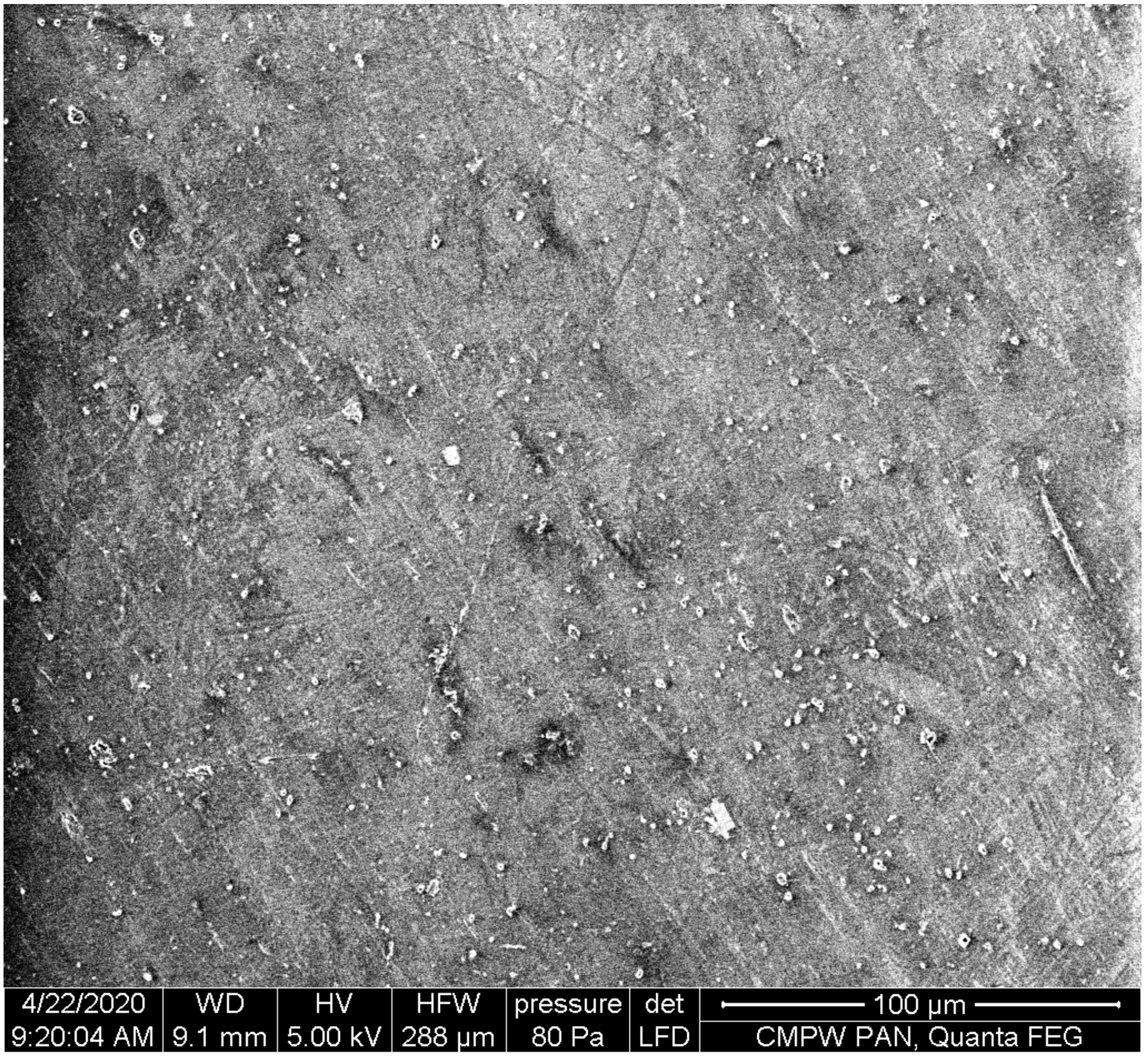

(**d**) SEM micrographs of the P(3HB-*co*-4HB)/30WF before degradation.

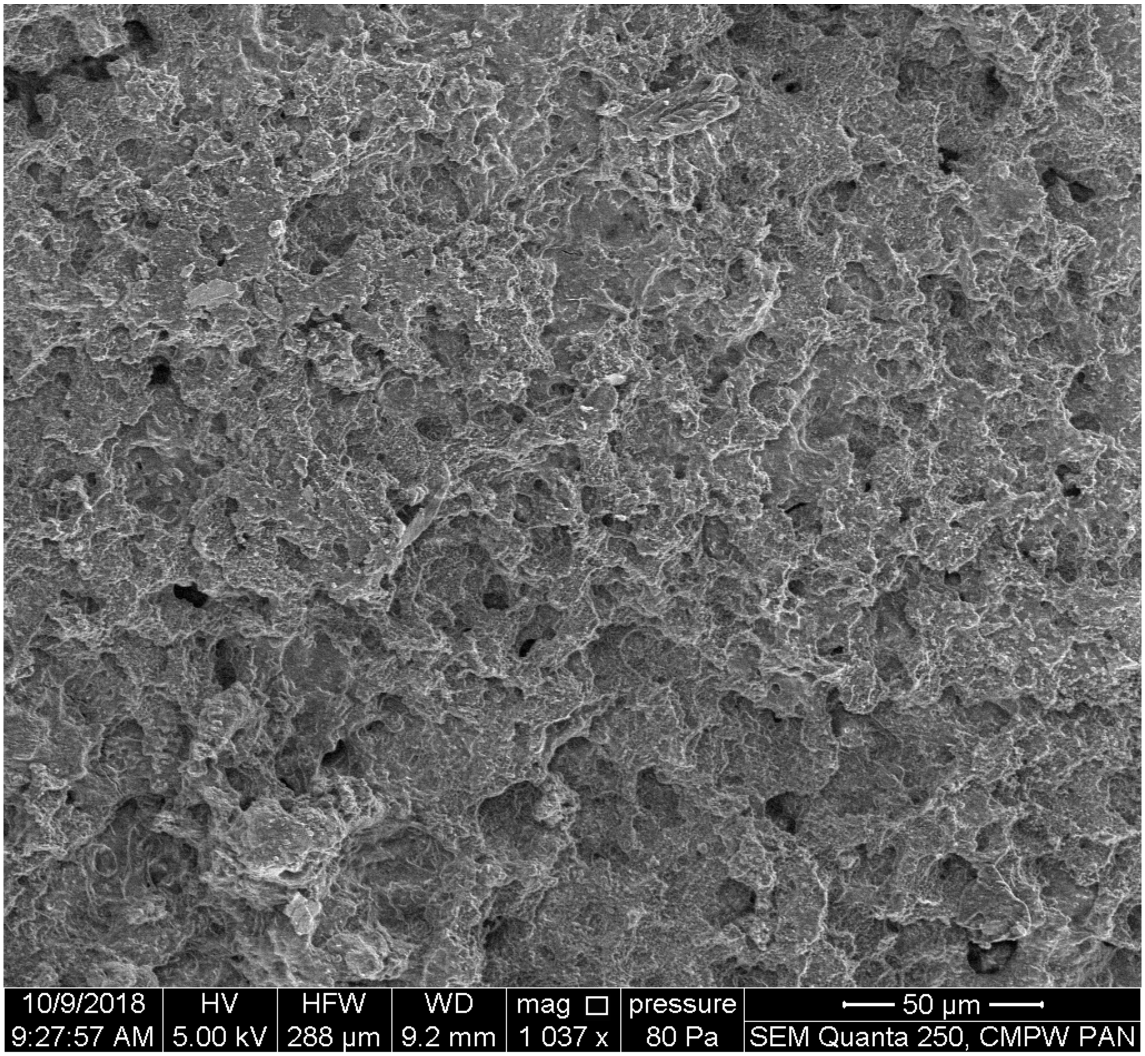

(**e**) SEM micrographs of the neat P(3HB-*co*-4HB) (100/0) after 21 days of degradation in BIODEGMA.

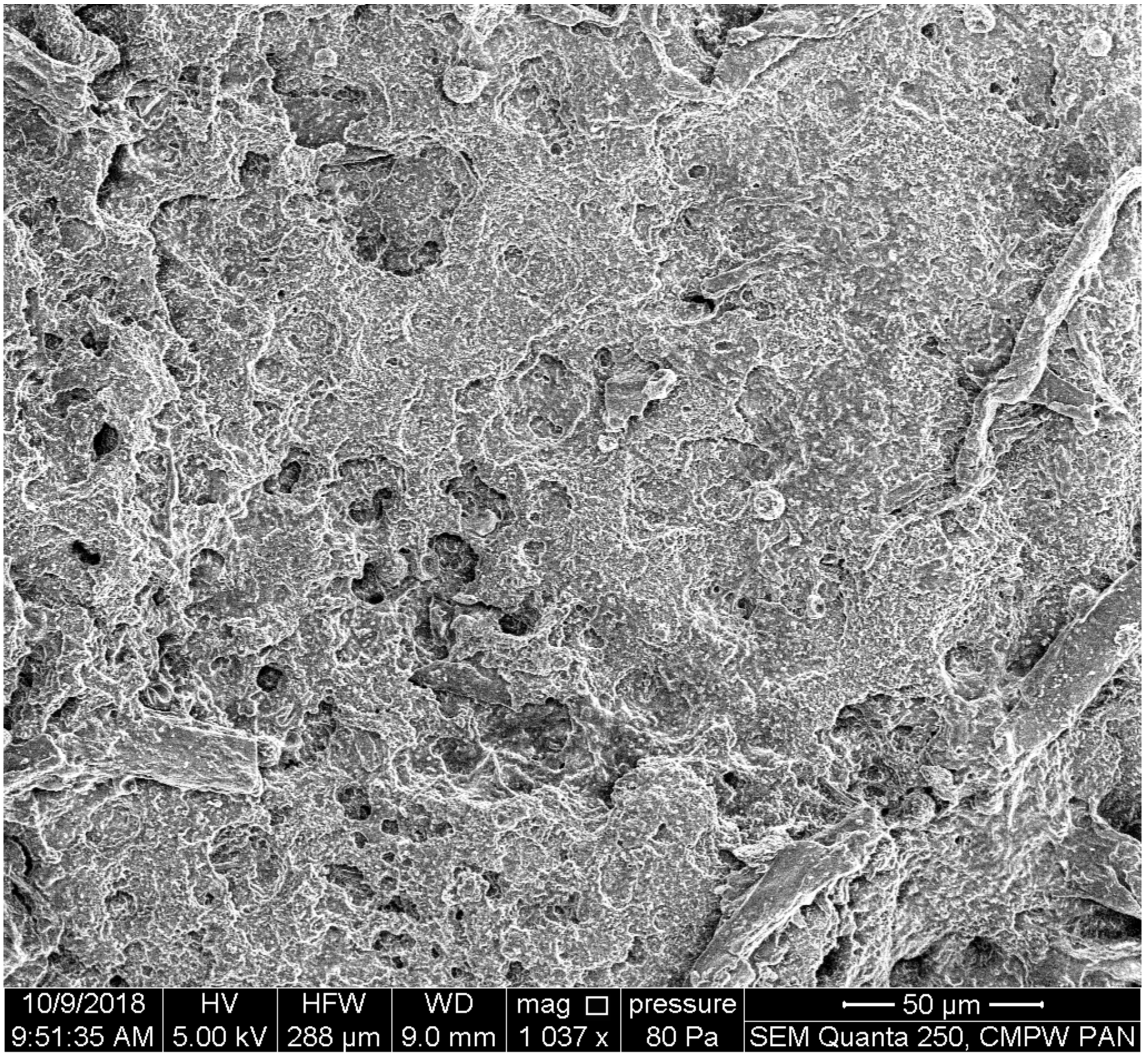

(**f**) SEM micrographs of the P(3HB-*co*-4HB)/10WF after 21 days of degradation in BIODEGMA.

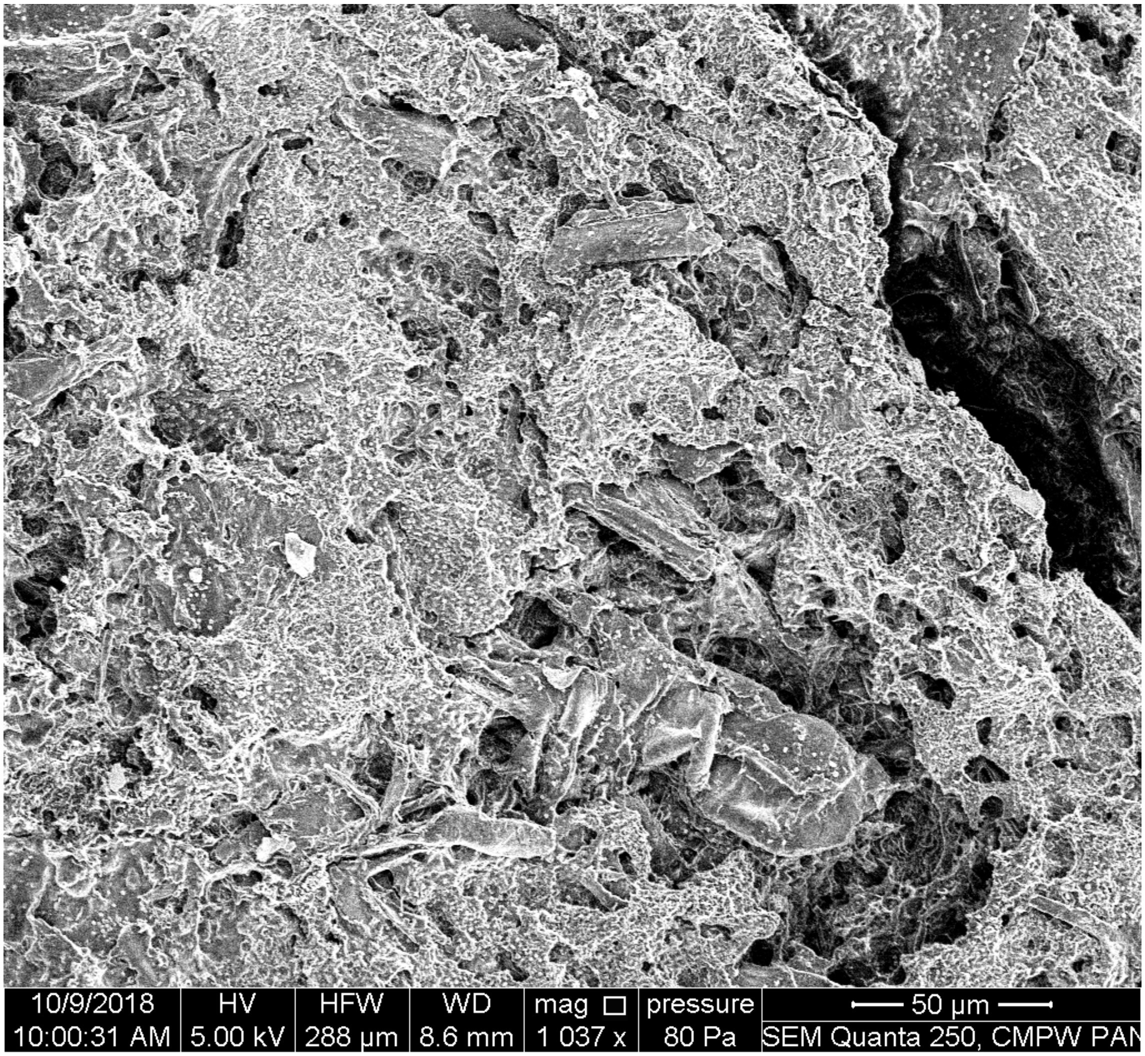

(**g**) SEM micrographs of the P(3HB-*co*-4HB)/20WF after 21 days of degradation in BIODEGMA.

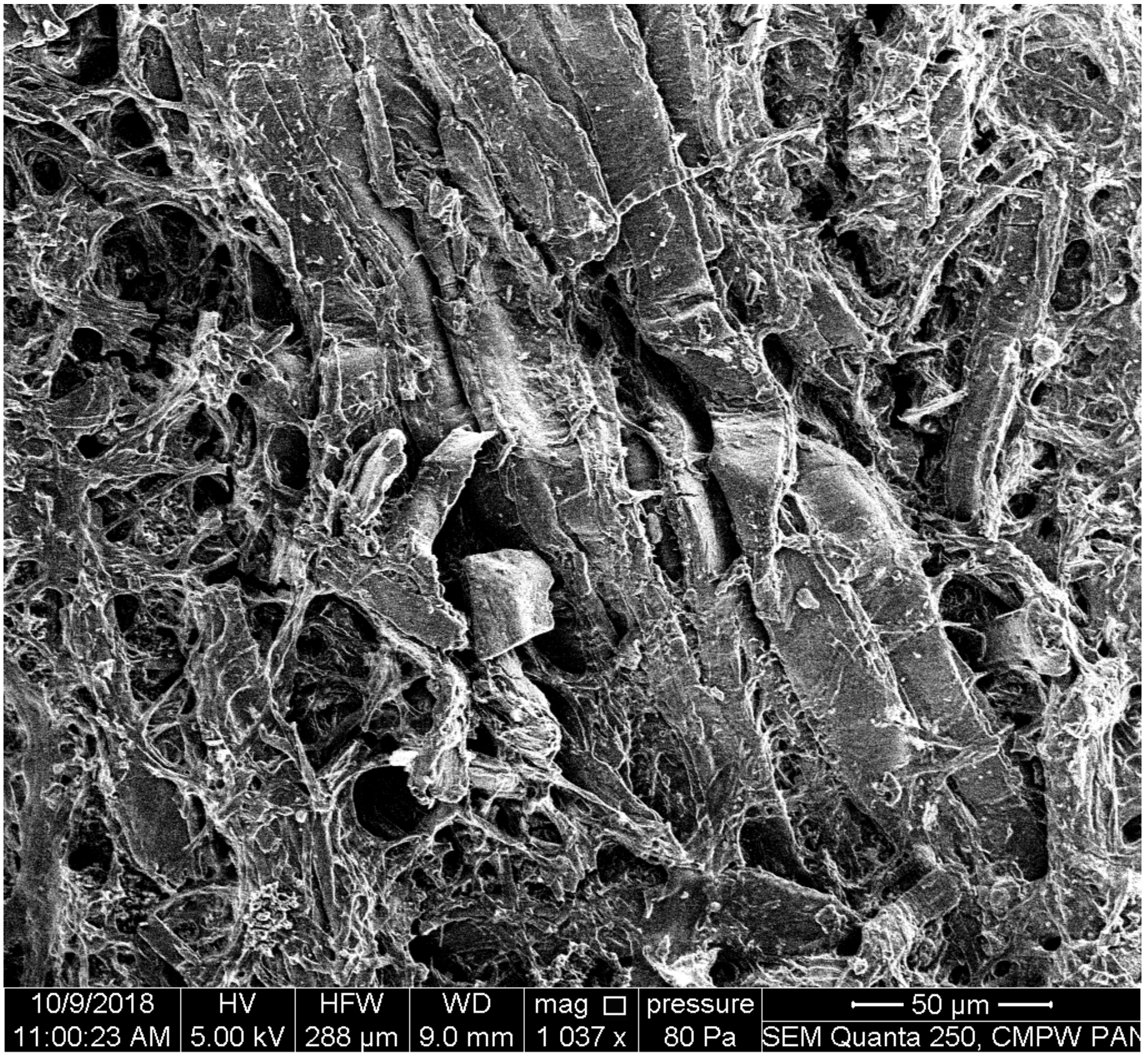

(**h**) SEM micrographs of the P(3HB-*co*-4HB)/30WF after 21 days of degradation in BIODEGMA.

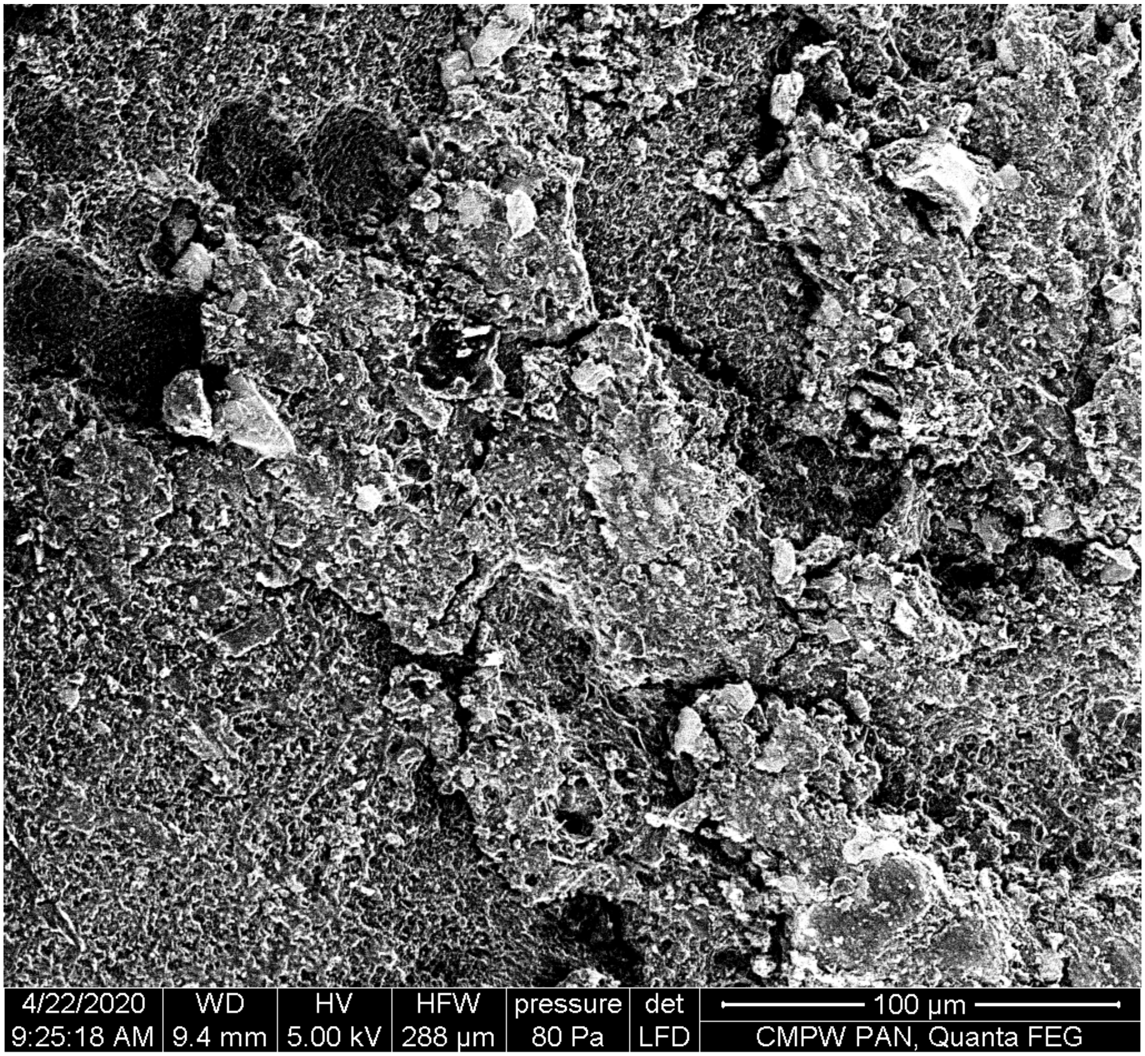

(**i**) SEM micrographs of the neat P(3HB-*co*-4HB) (100/0) after 21 days of degradation in Respirometer.

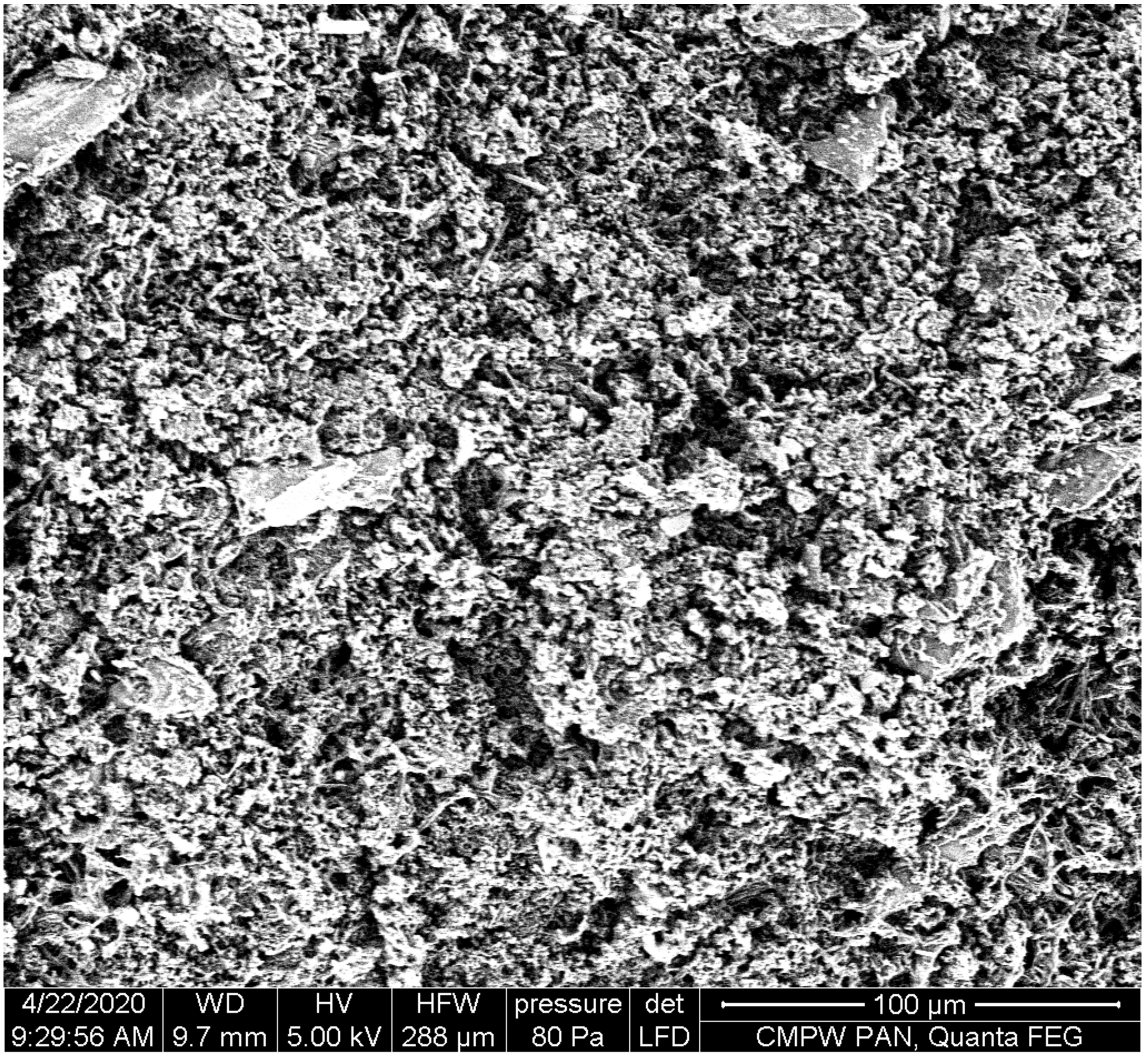

(**j**) SEM micrographs of the P(3HB-*co*-4HB)/10WF after 21 days of degradation in Respirometer

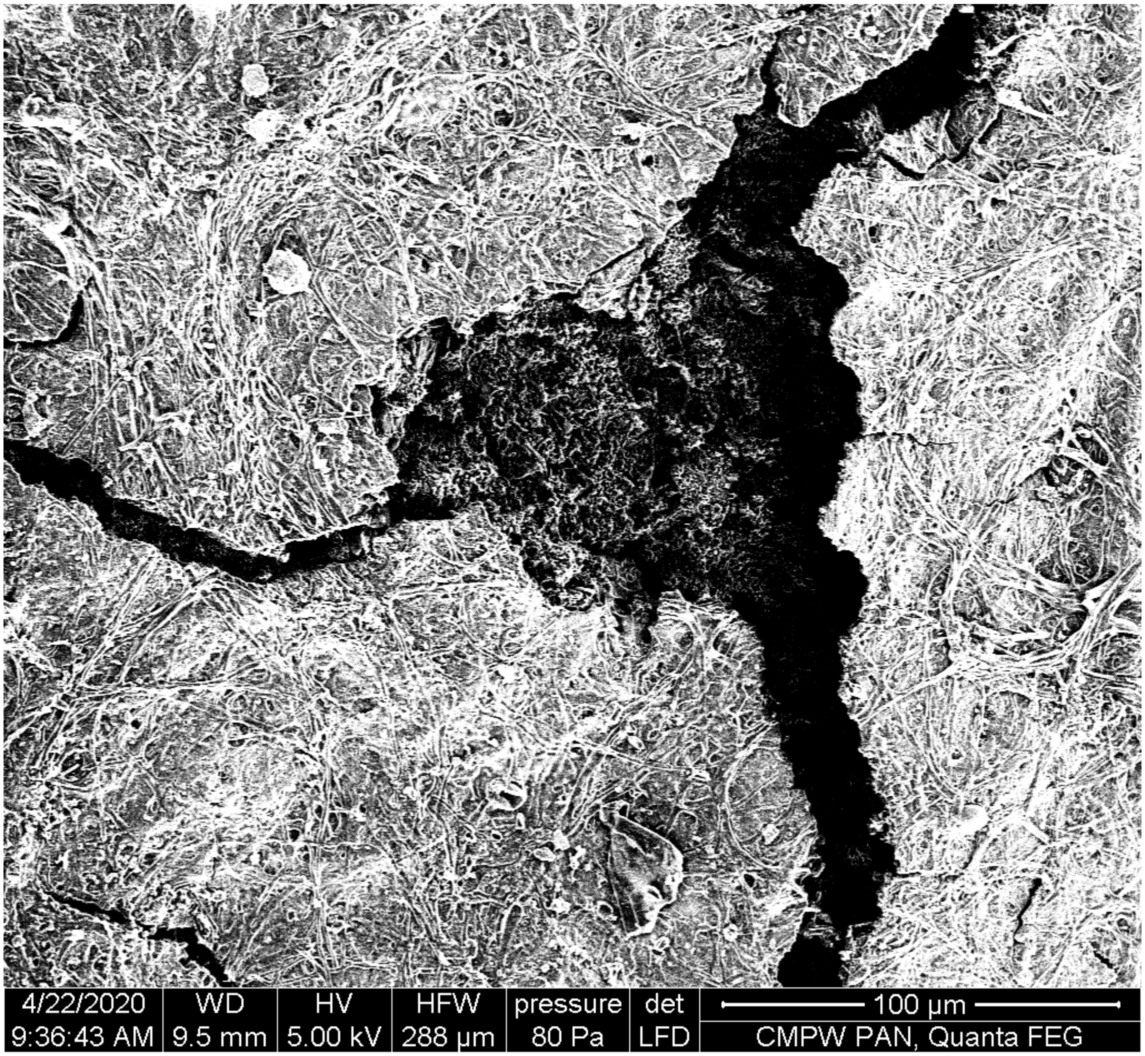

(**k**) SEM micrographs of the P(3HB-*co*-4HB)/20WF after 21 days of degradation in Respirometer.

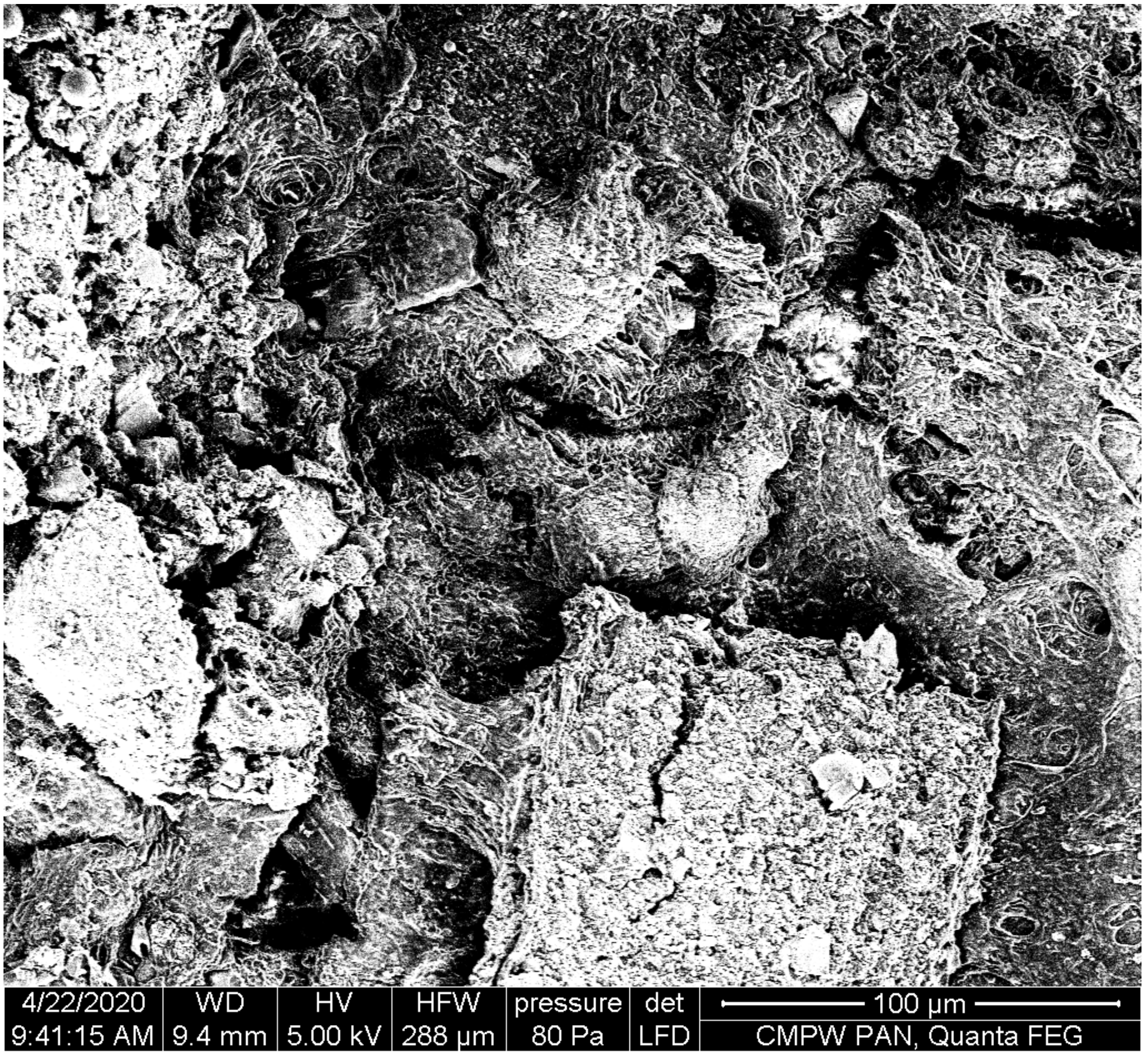

(**l**) SEM micrographs of the P(3HB-*co*-4HB)/30WF after 21 days of degradation in Respirometer.

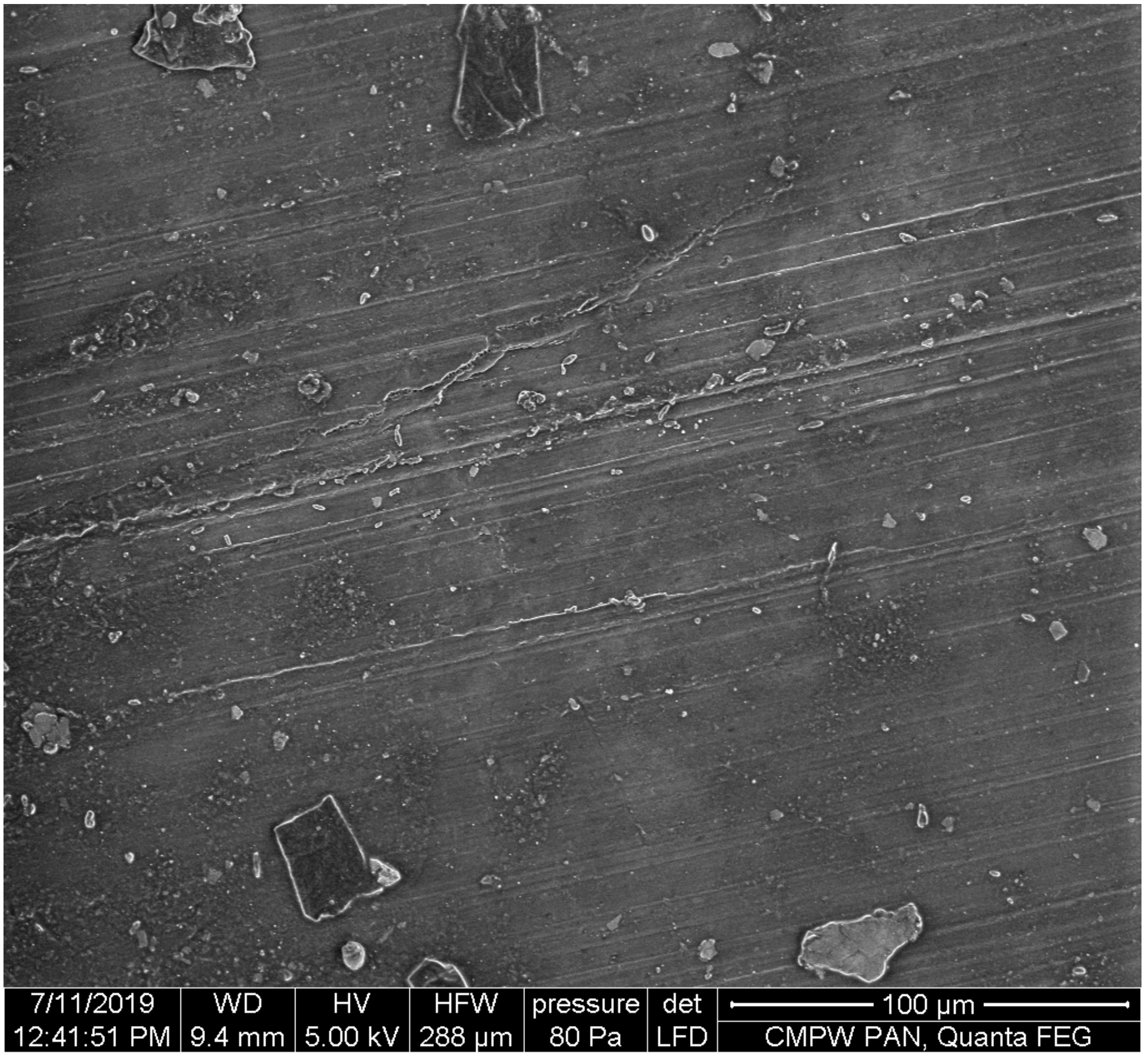

(**m**) SEM micrographs of the neat P(3HB-*co*-4HB) (100/0) after 21 days of degradation in water.

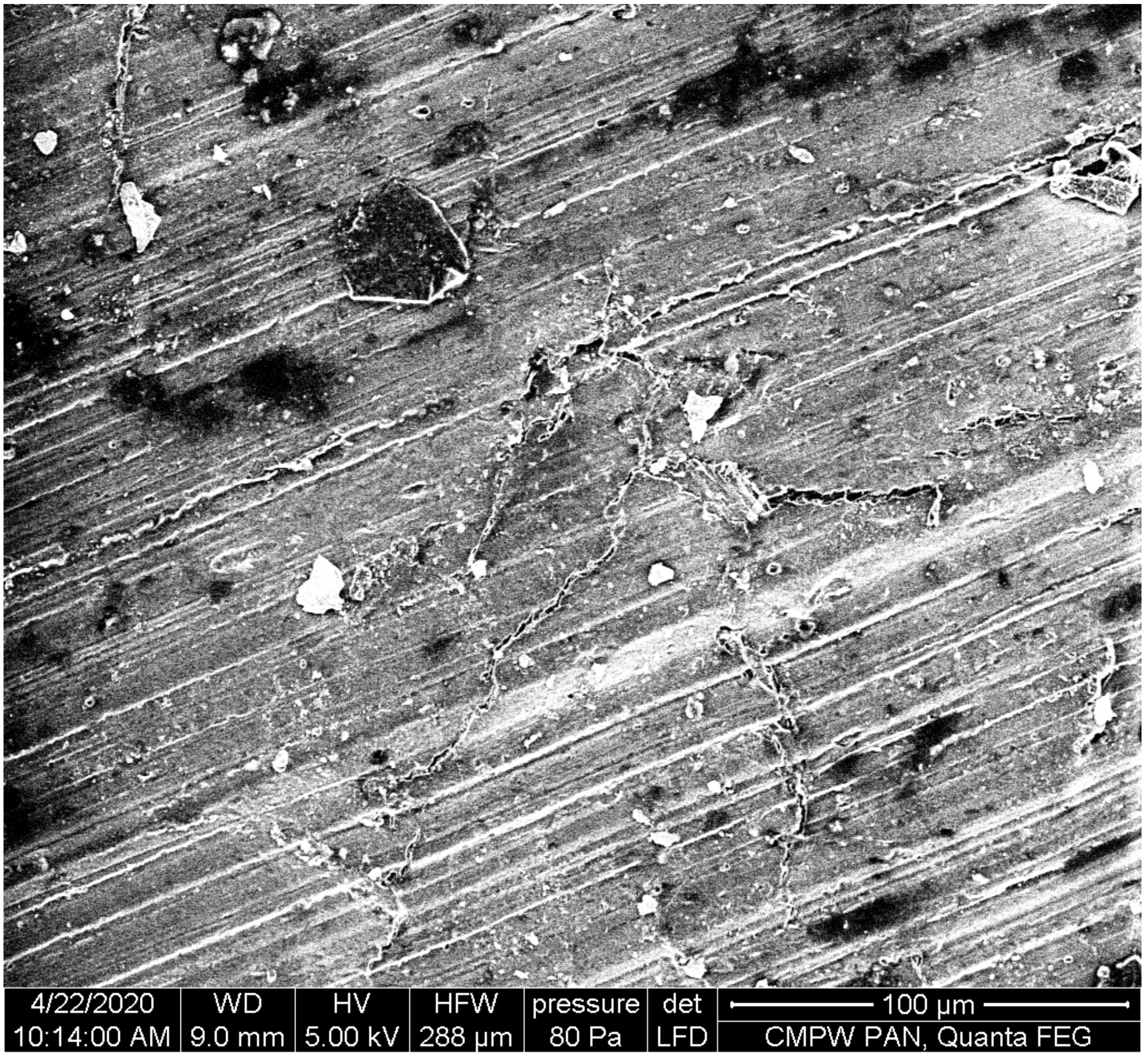

(**n**) SEM micrographs of the P(3HB-*co*-4HB)/10WF after 21 days of degradation in water.

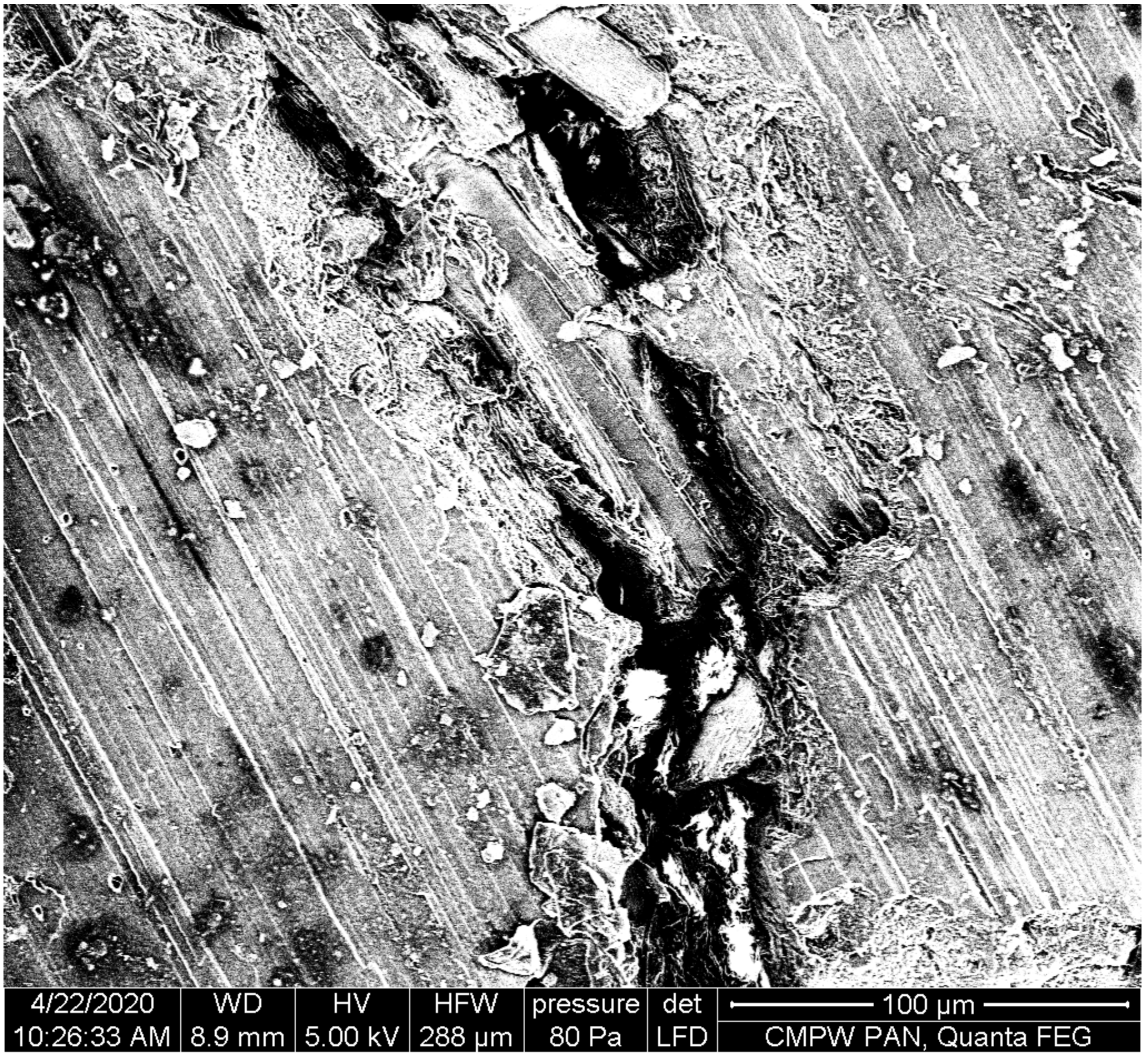

(**o**) SEM micrographs of the P(3HB-*co*-4HB)/20WF after 21 days of degradation in water.

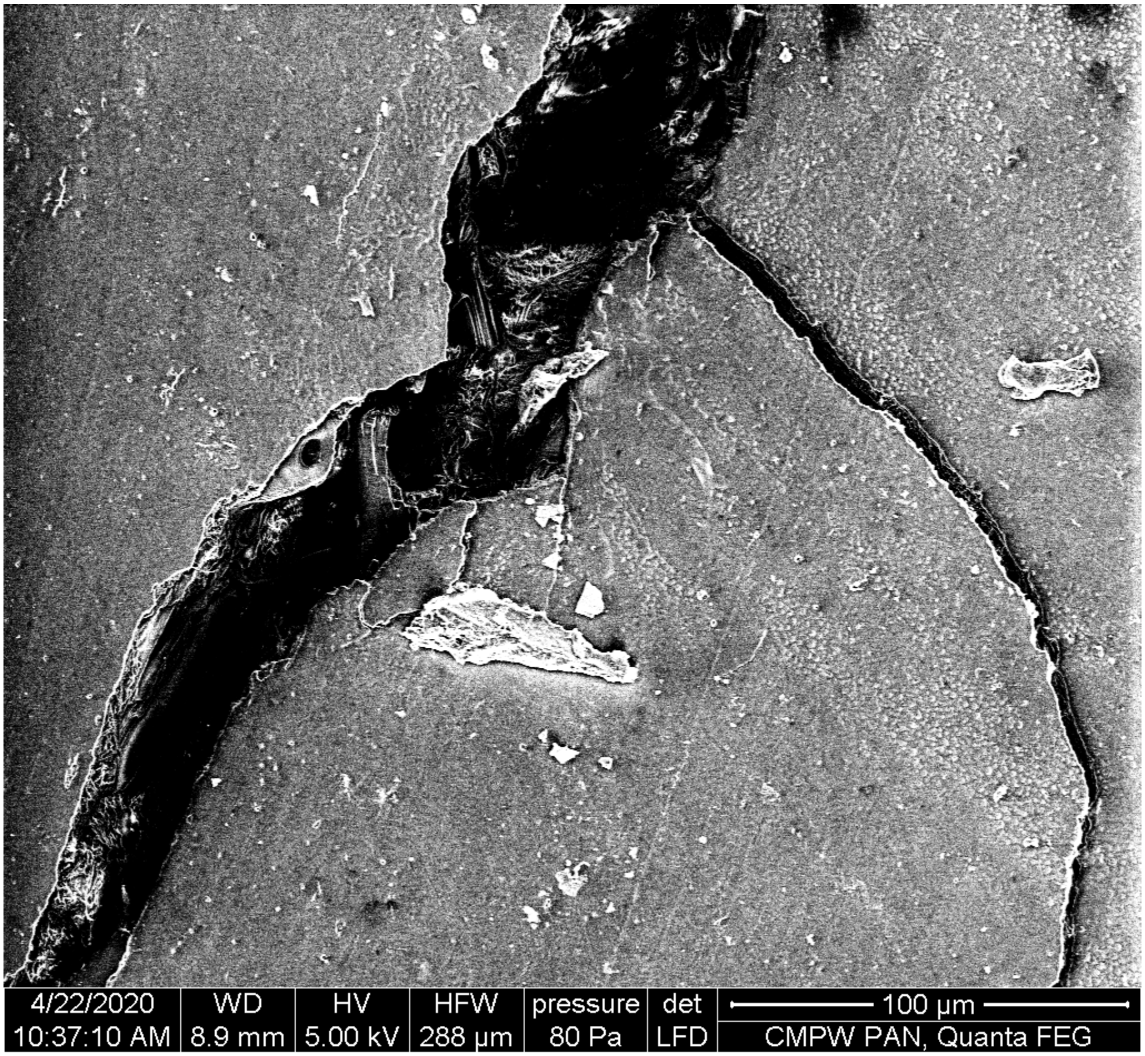

(**p**) SEM micrographs of the P(3HB-*co*-4HB)/30WF after 21 days of degradation in water.

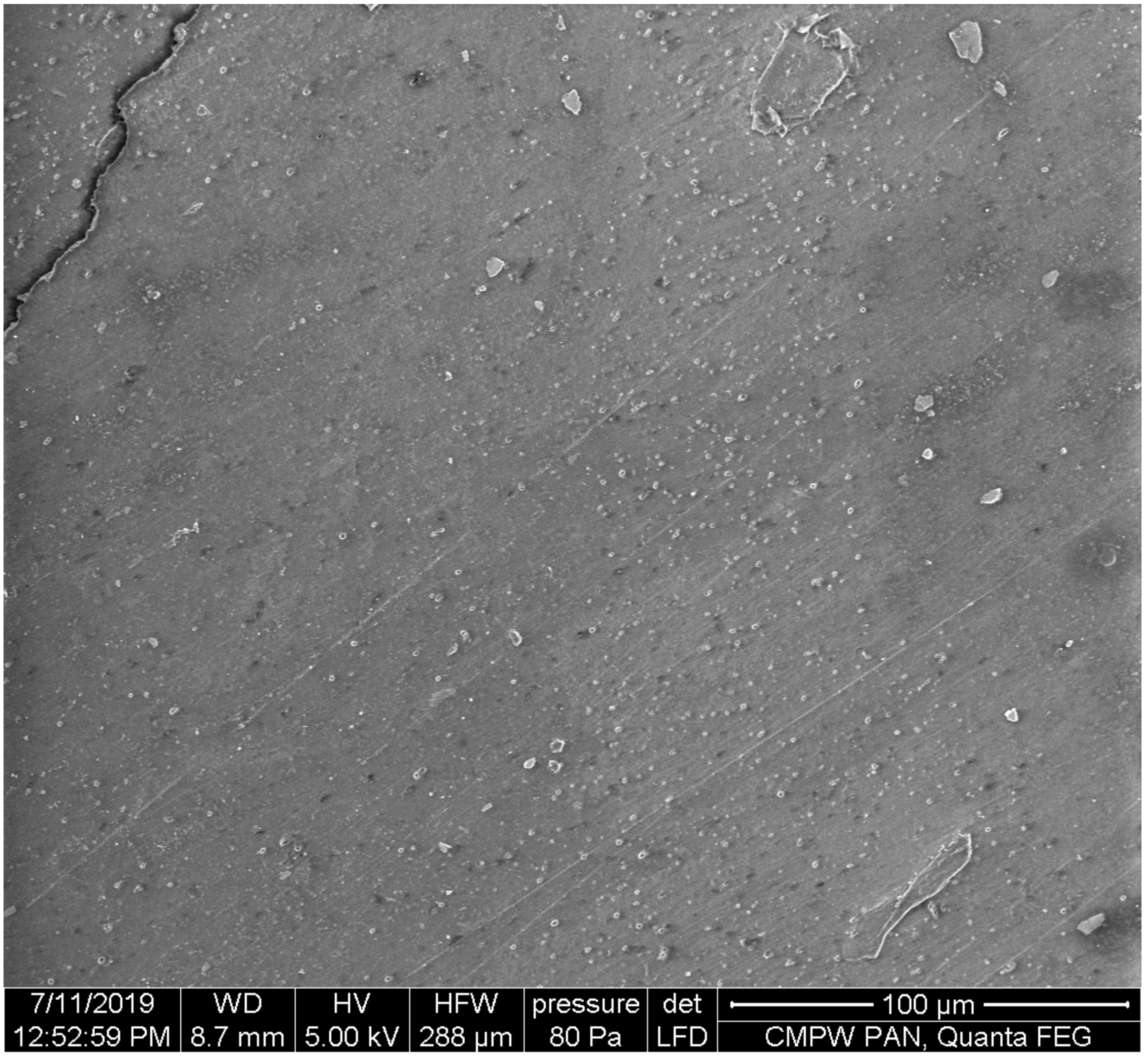

(**q**) SEM micrographs of the neat P(3HB-*co*-4HB) (100/0) after 21 days of degradation in buffer.

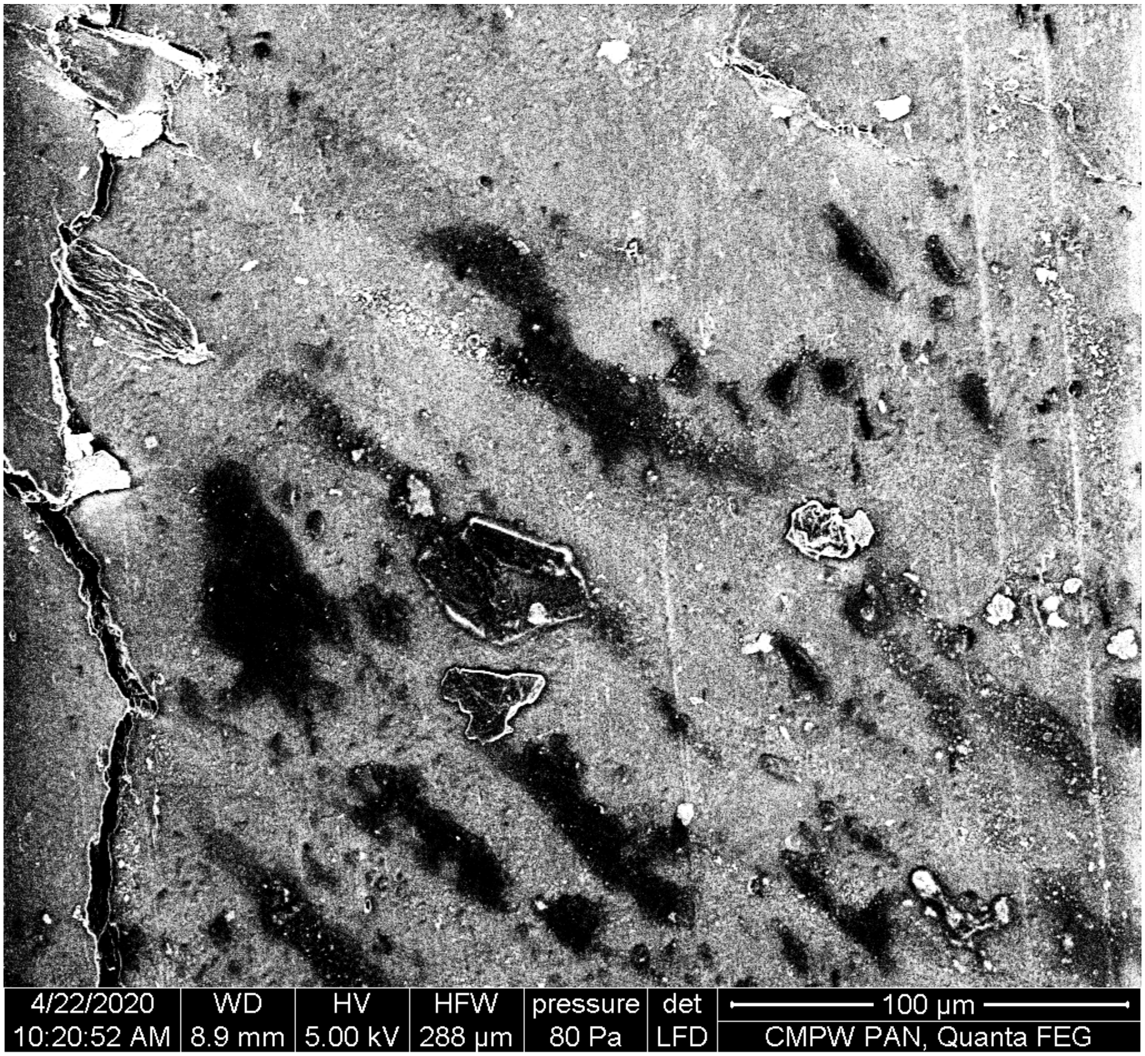

(**r**) SEM micrographs of the P(3HB-*co*-4HB)/10WF after 21 days of degradation in buffer.

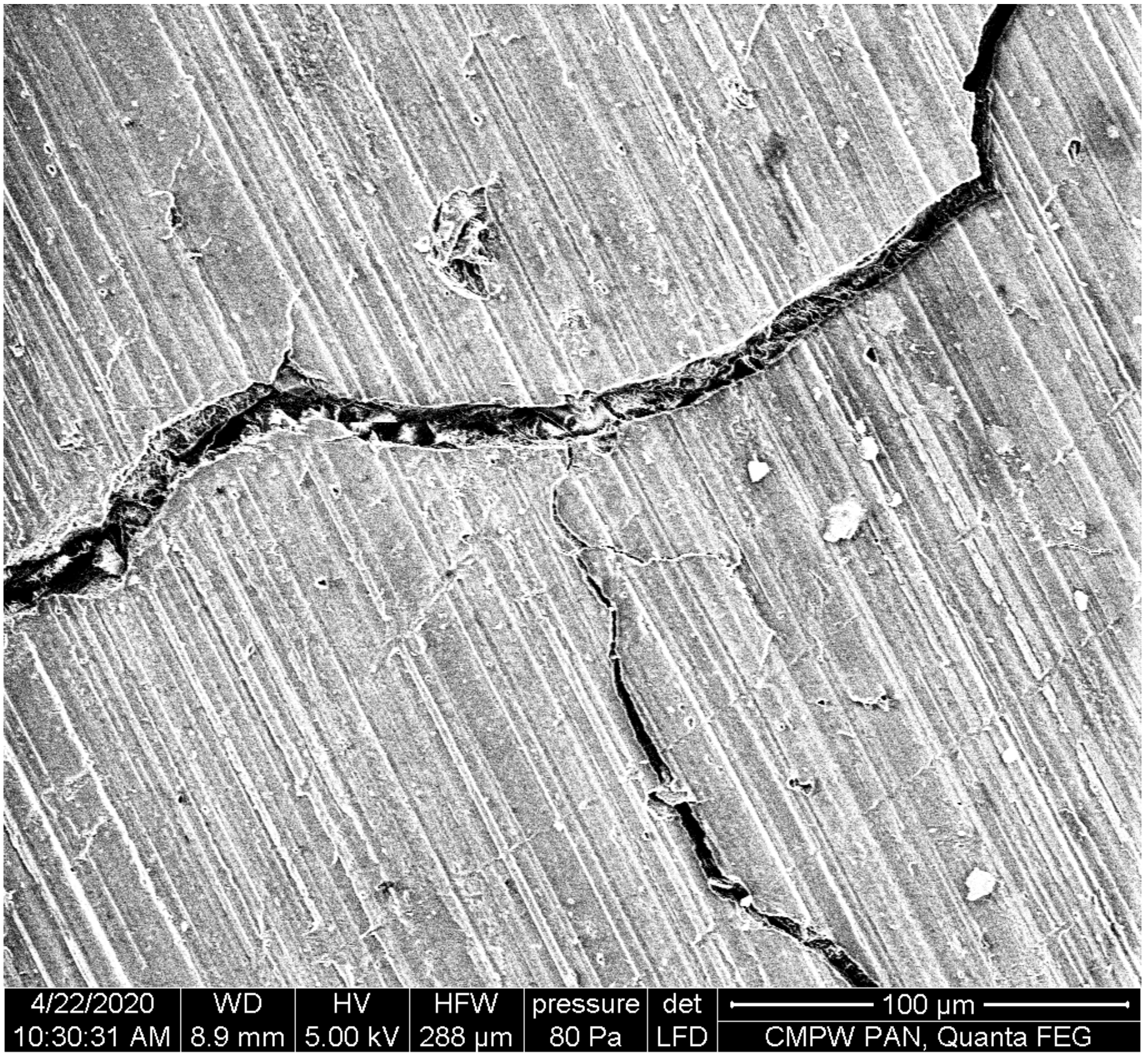

(**s**) SEM micrographs of the P(3HB-*co*-4HB)/20WF after 21 days of degradation in buffer.

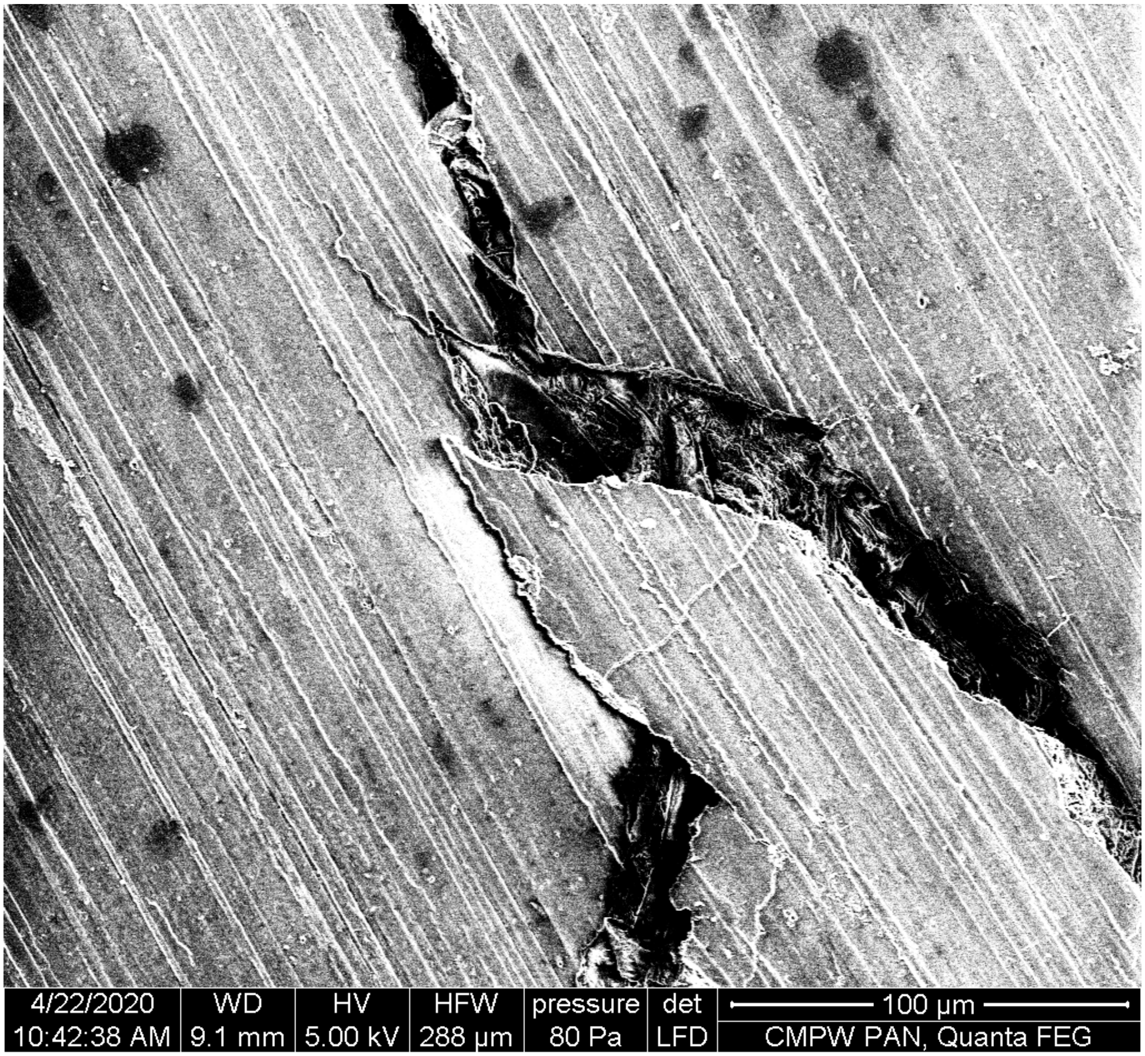

(**t**) SEM micrographs of the P(3HB-*co*-4HB)/30WF after 21 days of degradation in buffer.

Figure S1. SEM micrographs.

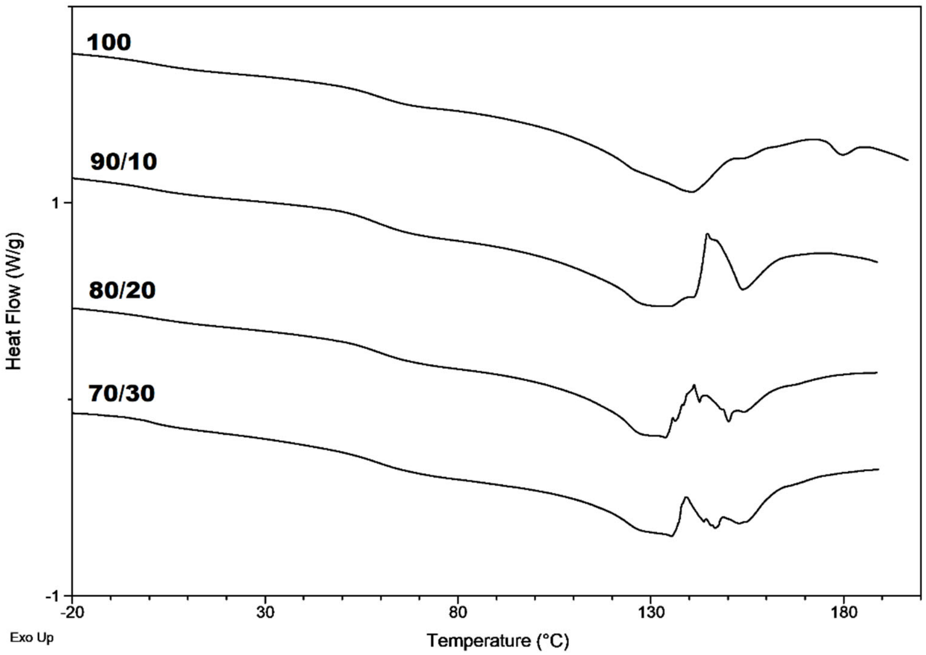

(**a**) DSC plot of neat P(3HB-*co*-4HB) (100/0) and P(3HB-*co*-4HB)/WF composites with the mass ratio of 90/10, 80/20 and 70/30 before degradation.

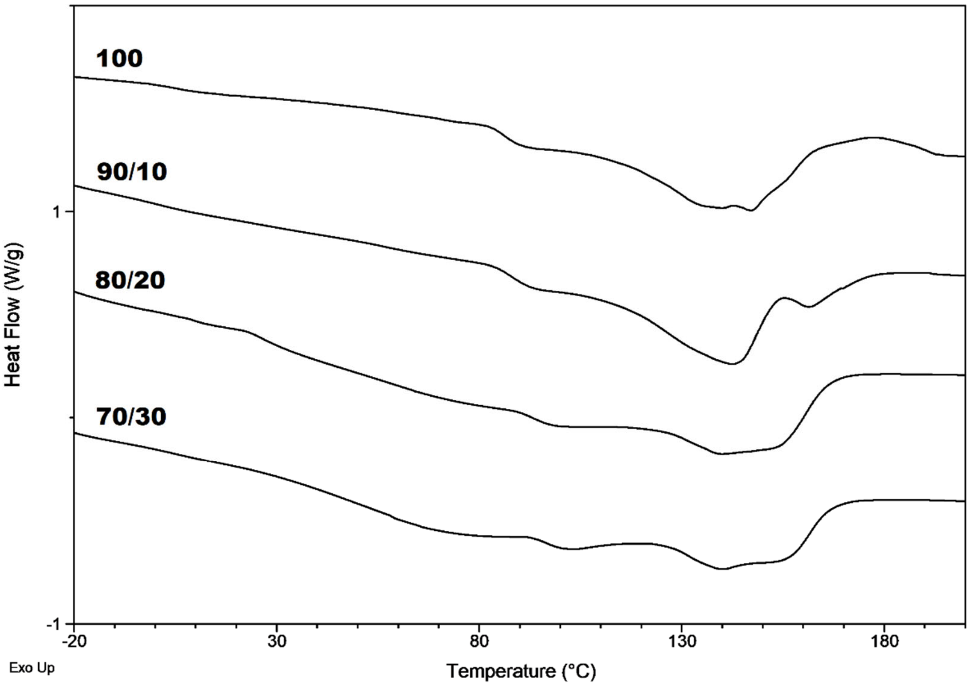

(**b**) DSC plot of neat P(3HB-*co*-4HB) (100/0) and P(3HB-*co*-4HB)/WF composites with the mass ratio of 90/10, 80/20 and 70/30 after 21 days of degradation in the BIODEGMA.

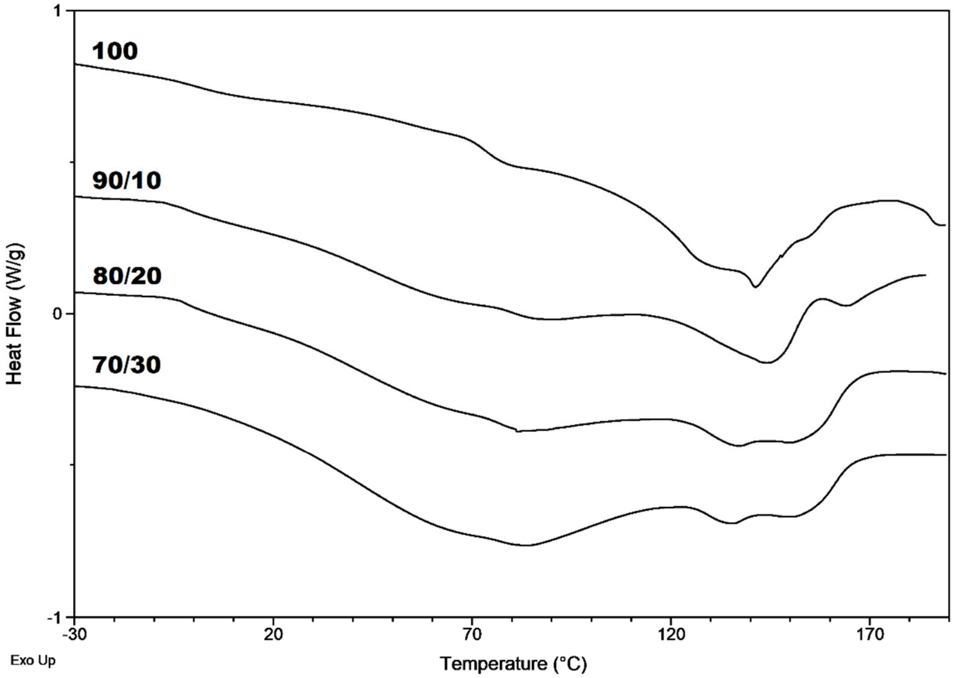

(**c**) DSC plot of neat P(3HB-*co*-4HB) (100/0) and P(3HB-*co*-4HB)/WF composites with the mass ratio of 90/10, 80/20 and 70/30 after 21 days of degradation in the respirometer.

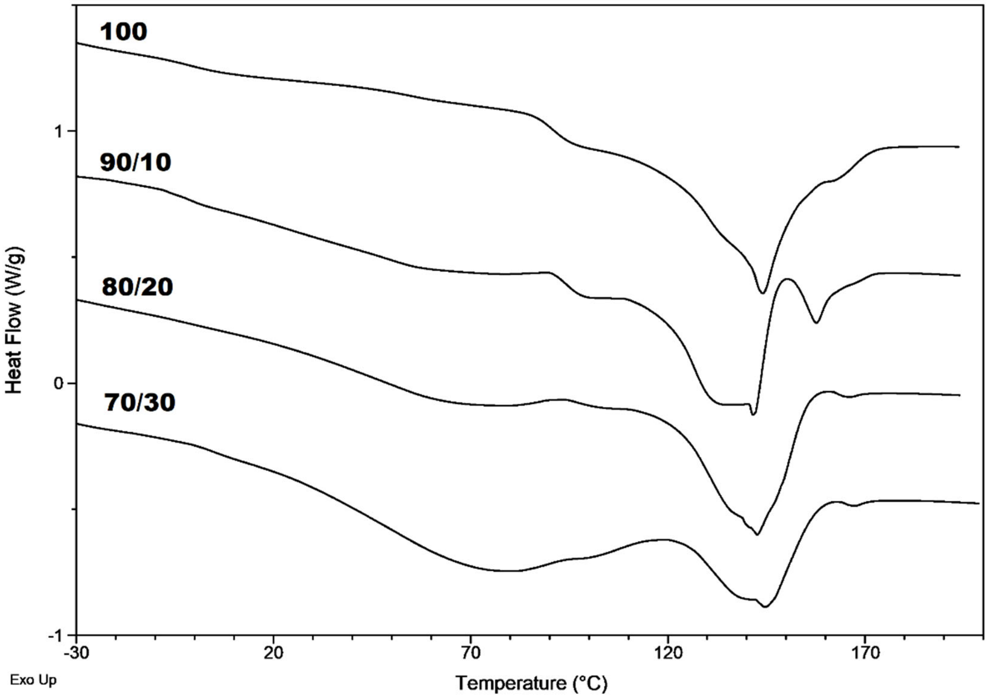

(**d**) DSC plot of neat P(3HB-*co*-4HB) (100/0) and P(3HB-*co*-4HB)/WF composites with the mass ratio of 90/10, 80/20 and 70/30 after 21 days of degradation in the water.

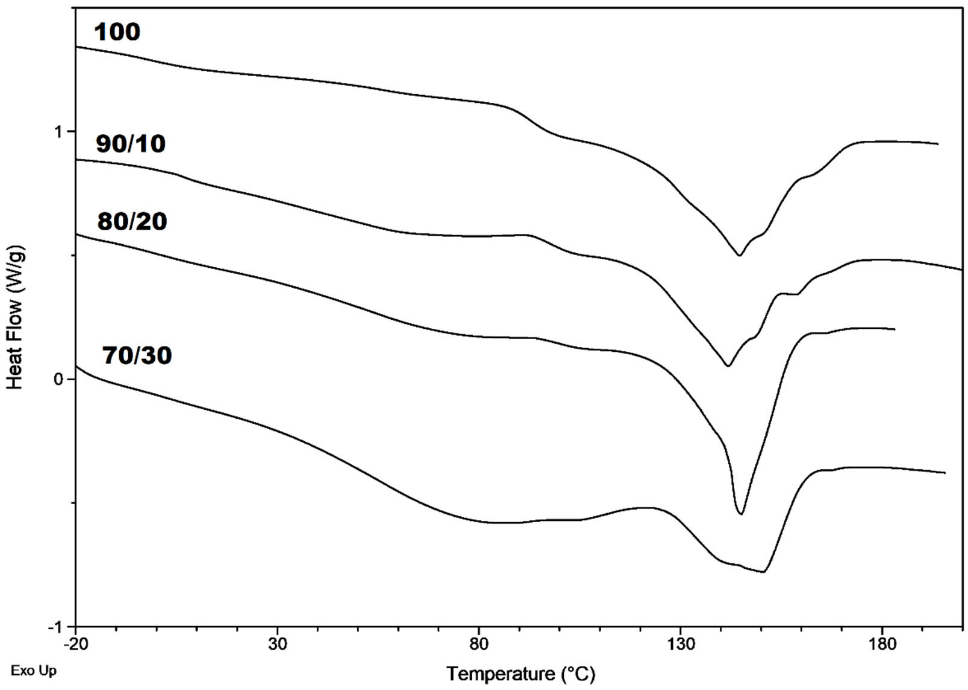

(**e**) DSC plot of neat P(3HB-*co*-4HB) (100/0) and P(3HB-*co*-4HB)/WF composites with the mass ratio of 90/10, 80/20 and 70/30 after 21 days of degradation in the buffer.

Figure 2. DSC plot.

© 2020 by the authors. Submitted for possible open access publication under the terms and conditions of the Creative Commons Attribution (CC BY) license (http://creativecommons.org/licenses/by/4.0/).
